# Unsaturated Gemini Surfactants with a Semi-Rigid Spacer: Micellization and Antimicrobial Activity

**DOI:** 10.3390/molecules31213969

**Published:** 2026-11-03

**Authors:** Iwona Kowalczyk, Adrianna Szulc, Justyna Brycka, Anna Komasa, Anna Koziróg, Bartosz Grabowski, Bogumił Brycki

**Affiliations:** 1 Department of Bioactive Products, Faculty of Chemistry, Adam Mickiewicz University Poznan, Uniwersytetu Poznańskiego 8, 61-614 Poznan, Poland; iwkow@amu.edu.pl (I.K.); aniak@amu.edu.pl (A.K.); 2 GEMSUR Sp. z o.o., Parkowa 6, Swadzim, 62-080 Tarnowo Podgorne, Poland; adrszu@gmail.com (A.S.); j.brycka@gemsur.com (J.B.); 3 Institute of Fermentation Technology and Microbiology, Faculty of Biotechnology and Food Sciences, Lodz University of Technology, 90-530 Lodz, Poland; anna.kozirog@p.lodz.pl (A.K.); bakagrabowski@gmail.com (B.G.)

**Keywords:** gemini surfactants, semi-rigid spacer, critical micelle concentration, antimicrobial activity, alkyl-chain length, cut-off effect, structure–property relationship

## Abstract

A homologous series of but-2-ene-1,4-bis(*N*-alkyl-*N*,*N*-dimethylammonium bromides) containing C12, C14, C16, and C18 alkyl chains was investigated to determine the influence of hydrophobic-chain length and a semi-rigid unsaturated spacer on aggregation and antimicrobial properties. The critical micelle concentration decreased from the C12 to the C16 derivative, whereas further chain elongation to C18 resulted in an increase in CMC, indicating that micellization is governed not only by hydrophobicity but also by molecular packing effects. Negative standard Gibbs energies confirmed spontaneous micellization. Antimicrobial activity showed the opposite trend: the C12 derivative was the most active compound against all tested microorganisms, with MIC (minimum inhibitory concentration) values as low as 0.06 mM (12-4ene-12 against *C. albicans*). Increasing alkyl-chain length markedly reduced activity, particularly against *Escherichia coli*, for which the MIC increased from 0.12 mM for C12 to over 62.5 mM for C18, demonstrating a pronounced cut-off effect. *Staphylococcus aureus* remained more susceptible to longer-chain homologues than *E. coli*, while notable activity was also observed against yeast and filamentous fungi. DFT calculations further indicated that spacer unsaturation affects the electronic properties of the molecules. Overall, the results demonstrate a strong relationship between alkyl-chain length, micellization, spacer rigidity, and antimicrobial performance.

## 1. Introduction

Gemini surfactants constitute a distinctive class of amphiphilic compounds composed of two hydrophobic chains and two hydrophilic head groups linked by a molecular spacer [1,2,3,4]. Compared with conventional monomeric surfactants, this dimeric architecture generally provides enhanced surface activity, lower critical micelle concentrations, and a pronounced tendency toward self-assembly. These properties have stimulated considerable interest in gemini surfactants as functional materials for applications in detergency, cosmetics, pharmaceutical formulations, antimicrobial systems, nanotechnology, and other fields in which efficient interfacial activity and molecular organization are required [5,6,7,8,9,10].

Among gemini surfactants, bis-quaternary ammonium compounds are of particular interest because they combine strong surface activity with the biological properties characteristic of cationic amphiphiles. Their physicochemical behavior is strongly governed by molecular architecture, including the length of the hydrophobic chains, the chemical nature and length of the spacer, the structure of the cationic head groups, and the type of counterion [11,12,13,14,15,16]. Even relatively small structural modifications may significantly affect adsorption at interfaces, micellization, counterion binding, aggregate morphology, and interactions with biological membranes. Consequently, systematic studies based on homologous series are particularly useful for establishing relationships between molecular structure and physicochemical or biological properties.

The spacer connecting the two quaternary ammonium groups plays an especially important role in determining the conformation and intermolecular organization of gemini surfactants. Flexible aliphatic or heteroatom-containing spacers permit considerable conformational freedom, whereas more rigid spacers may impose a defined separation and orientation of the charged head groups [17,18,19]. Introduction of a carbon–carbon double bond into the spacer decreases rotational freedom and increases conformational rigidity [5]. In contrast to classical saturated polymethylene spacers, which allow relatively free rotation around successive C–C single bonds, the but-2-ene spacer contains a central C=C double bond that restricts rotational freedom and reduces conformational flexibility. Such structural restriction may influence both packing at the interface and organization within micellar aggregates, and may also affect the ability of the surfactant molecule to interact with microbial membranes. Nevertheless, unsaturated gemini surfactants containing a short but-2-ene spacer have been investigated much less extensively than many classical polymethylene-linked homologues.

**Figure 1:**
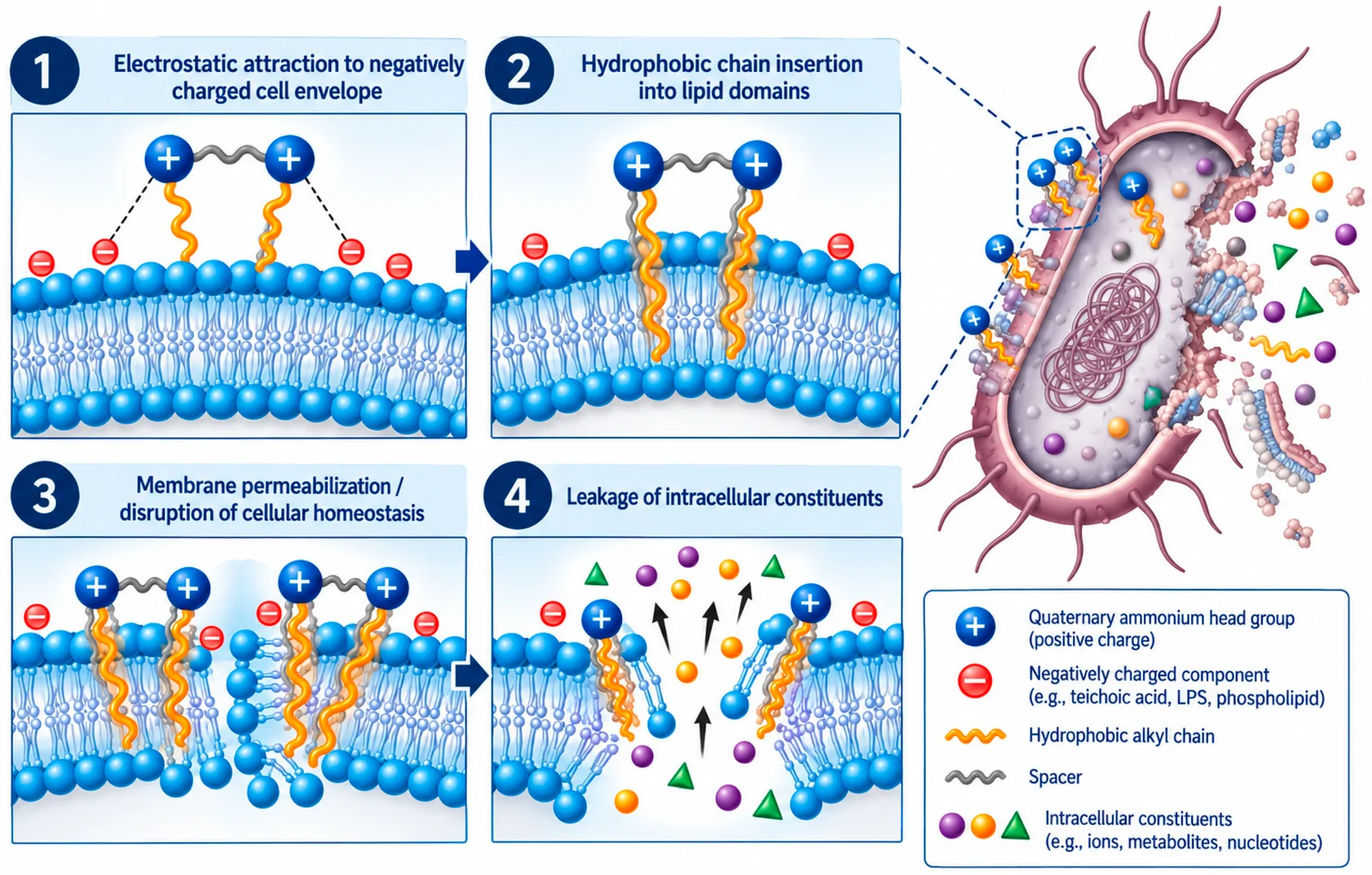
General schematic representation of a proposed mechanism of antimicrobial action of quaternary ammonium gemini surfactants. (1) Electrostatic attraction between the positively charged quaternary ammonium head groups and negatively charged components of the microbial cell envelope; (2) insertion of hydrophobic alkyl chains into lipid domains of the membrane; (3) membrane permeabilization and disruption of cellular homeostasis; and (4) leakage of intracellular constituents, ultimately resulting in loss of cell viability.

An additional characteristic of quaternary ammonium gemini surfactants is their antimicrobial activity. Their mode of action is primarily associated with electrostatic attraction between positively charged ammonium groups and negatively charged components of microbial cell envelopes, followed by interaction of the hydrophobic chains with lipid domains of the membrane. This may lead to membrane permeabilization, disruption of cellular homeostasis, leakage of intracellular constituents, and ultimately loss of cell viability (Figure 1) [20,21,22,23,24]. The efficiency of this process is strongly dependent on hydrophobic chain length. In homologous series, antimicrobial activity frequently increases up to an optimum chain length and subsequently decreases for longer homologues, a phenomenon commonly referred to as the cut-off effect [25,26]. In addition to determining minimum inhibitory concentrations, monitoring bacterial growth over time provides further insight into the concentration- and inoculum-dependent inhibitory effects of antimicrobial compounds.

This structure-dependent behavior is particularly relevant for gemini surfactants because elongation of the alkyl chains simultaneously increases hydrophobicity and enhances the tendency toward aggregation. At sufficiently long chain lengths, strong self-association may reduce the concentration of free surfactant monomers available for interaction with microbial cell envelopes. Moreover, the susceptibility of microorganisms depends on the architecture of their cell walls and membranes. Gram-negative bacteria possess an additional lipopolysaccharide-rich outer membrane that can limit penetration of hydrophobic or strongly aggregated cationic compounds, whereas Gram-positive bacteria lack this outer barrier. Fungal cells, in turn, possess structurally distinct membranes and cell walls, and their response to cationic surfactants may therefore differ substantially from that of bacterial cells [27,28,29,30,31].

**Figure 2:**
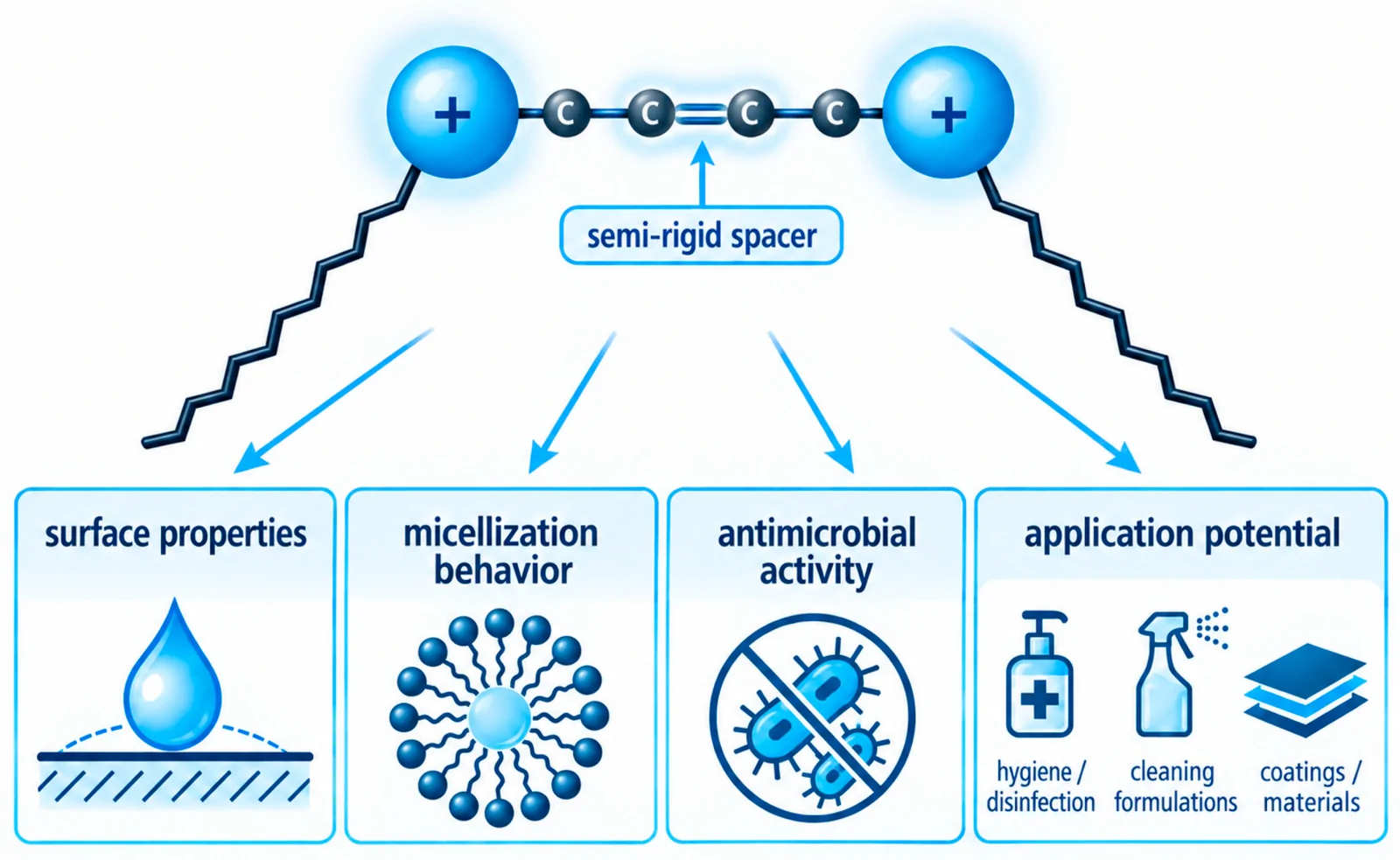
Schematic representation of a gemini surfactant with a semi-rigid C=C-containing spacer and its potential impact on interfacial behavior, self-assembly, antimicrobial activity, and possible applications.

Although quaternary ammonium-based gemini surfactants have been extensively investigated and numerous studies have described the influence of alkyl-chain length, spacer length, counterion type, and head-group structure on their physicochemical and biological properties, considerably less attention has been paid to gemini surfactants containing an unsaturated spacer. In particular, reports on compounds incorporating a carbon–carbon double bond directly within the spacer are scarce. The presence of the C=C bond restricts rotational freedom and reduces the conformational flexibility of the linker, so that such a spacer can be regarded as semi-rigid. This structural feature may influence the relative orientation and separation of the quaternary ammonium head groups, as well as molecular packing at interfaces and within supramolecular aggregates. Consequently, incorporation of a semi-rigid unsaturated spacer may have a significant impact on micellization, interfacial behavior, and interactions with biological membranes (Figure 2) [32,33,34,35]. Although related bis(alkylammonium) surfactants with unsaturated spacers have previously been reported as chloride salts [34], the present study concerns the corresponding bromide series, which represents a distinct homologous system. Nevertheless, systematic studies on homologous series of gemini quaternary ammonium surfactants containing a but-2-ene spacer remain limited.

The aim of this study was therefore to establish relationships between molecular structure, micellization behavior, and antimicrobial activity within a homologous series of unsaturated gemini quaternary ammonium salts. By maintaining an identical but-2-ene spacer and systematically varying the alkyl-chain length, the study provides insight into how hydrophobicity and molecular organization determine the functional properties of these compounds. The synthesized surfactants were characterized by ^1^H NMR, ^13^C NMR, and FTIR spectroscopy, and their aggregation behavior was assessed by conductometric determination of the critical micelle concentration and related thermodynamic parameters. Antimicrobial activity was evaluated by determining the minimum inhibitory concentrations against selected bacterial and fungal strains, while the effects of surfactant concentration and initial inoculum density on *Escherichia coli* growth were investigated through growth-kinetic measurements. Furthermore, quantum chemical calculations were performed to examine the influence of spacer unsaturation on the electronic properties of the surfactants.

## 2. Results and Discussion

### 2.1. Synthesis and Analysis

**Figure 3:**
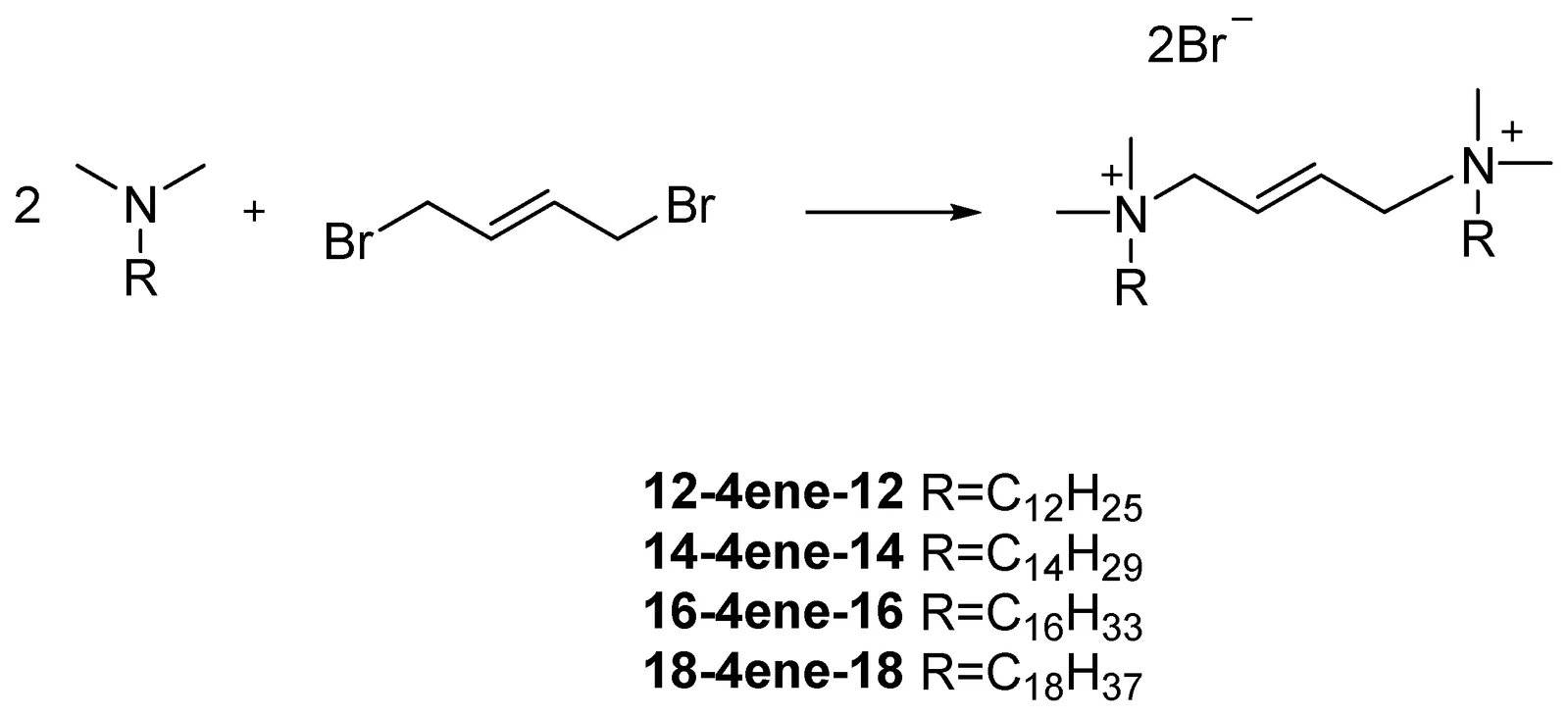
Synthesis of but-2-ene-1,4-bis(*N*-alkyl-*N*,*N*-dimethylammonium bromides).

A homologous series of but-2-ene-1,4-bis(*N*-alkyl-*N*,*N*-dimethylammonium bromides) was synthesized by the quaternization of 1,4-dibromobut-2-ene with the corresponding *N*-alkyl-*N*,*N*-dimethylamines (Figure 3). The investigated compounds contained two hydrophobic alkyl chains and an identical four-carbon spacer incorporating a carbon–carbon double bond, while the alkyl-chain length varied systematically from C12 to C18 across the homologous series. In each synthesis, 1 equivalent of 1,4-dibromobut-2-ene was reacted with 2 equivalents of the appropriate tertiary amine, namely *N*-dodecyl-*N*,*N*-dimethylamine, *N*-tetradecyl-*N*,*N*-dimethylamine, *N*-hexadecyl-*N*,*N*-dimethylamine, or *N*-octadecyl-*N*,*N*-dimethylamine. In this way, a series of unsaturated gemini surfactants containing C12, C14, C16, and C18 hydrocarbon chains was obtained.

The syntheses were performed in acetonitrile under reflux conditions at the boiling point of the solvent, with continuous stirring using a magnetic stirrer [36,37]. Acetonitrile was selected as a polar aprotic solvent suitable for quaternization reactions involving tertiary amines and alkyl halides. Under these conditions, the reaction mixture gradually changed its appearance and consistency as the reaction progressed. Solidification of the reaction mixture was used as a practical endpoint of the reaction. Under the applied conditions, progressive precipitation of the ionic product ultimately filled the reaction vessel and prevented further efficient stirring. Based on our experience with this homologous series, this behavior was reproducible for the investigated compounds. The reaction times and isolated yields were as follows: 12-4ene-12: [9 h], [97%]; 14-4ene-14: [8 h], [96%]; 16-4ene-16: [7 h], [96%]; and 18-4ene-18: [4 h], [95%]. A clear relationship between the alkyl chain length of the tertiary amine and the reaction time was observed. The reaction time progressively decreased with increasing length of the hydrocarbon chain, with the C18 derivative being formed in the shortest time among the investigated homologues. Despite the differences in reaction time, the syntheses were highly efficient, and the isolated yields exceeded 95% for all synthesized compounds.

The formation of the gemini quaternary ammonium salts proceeds through the Menshutkin quaternization reaction. The process involves nucleophilic substitution of the bromide-bearing carbon atoms of 1,4-dibromobut-2-ene by the nitrogen atoms of two tertiary amine molecules. The reaction can therefore be described as an S_N_2-type nucleophilic substitution, leading to the formation of two quaternary ammonium centers within a single molecule [5]. Both positively charged nitrogen atoms are connected through a four-carbon spacer containing a central C=C double bond, while the two long alkyl substituents form the hydrophobic parts of the resulting gemini surfactants. The applied synthetic strategy enables the preparation of structurally analogous compounds differing only in the length of their hydrophobic chains, which is particularly useful for investigating structure–property relationships within the homologous series.

After completion of the reaction, the crude products were subjected to purification by crystallization from an acetonitrile/methanol mixture (10:1, *v*/*v*). The purified products were subsequently dried at 60 °C and then additionally dried over P_4_O_10_ under vacuum in a desiccator to remove residual solvent and moisture. The obtained compounds were isolated as solid products suitable for further physicochemical and biological studies.

The described procedure provided a convenient and reproducible route for the preparation of unsaturated gemini quaternary ammonium compounds. The combination of high isolated yields, straightforward purification, and the possibility of obtaining a homologous series under comparable reaction conditions makes this synthetic approach particularly suitable for systematic studies of the influence of alkyl chain length on the physicochemical and biological properties of gemini surfactants.

The synthesized compounds were obtained as solids and exhibited relatively high melting points, characteristic of ionic gemini quaternary ammonium salts. The melting points of the C12, C14, C16, and C18 derivatives were 216–217, 218–220, 216–218, and 178–180 °C, respectively. The C12–C16 homologues showed comparable thermal behavior, whereas a pronounced decrease in melting point was observed for the C18 derivative. This decrease may be related to changes in crystal packing and intermolecular interactions associated with the increased length and conformational flexibility of the hydrophobic alkyl chains.

The structures of the synthesized gemini surfactants were confirmed by ^1^H NMR, ^13^C NMR, and FTIR spectroscopy. Proton and carbon chemical shifts are listed in Table 1 and Table 2, respectively. For all synthesized compounds, the vicinal coupling constant of the olefinic protons was approximately 14–16 Hz, which is consistent with and confirms the trans configuration of the C=C bond in the products.

Fourier Transform Infrared Spectroscopy (FTIR) spectra of but-2-ene-1,4-bis(*N*-alkyl-*N*,*N*-dimethylammonium bromides) exhibit the characteristic absorption bands associated with the asymmetric and symmetric stretching vibrations of the aliphatic CH_3_ and CH_2_ groups, together with the corresponding deformation modes. A characteristic band observed at approximately 952–970 cm^−1^ can be attributed to the out-of-plane deformation vibration of the trans –CH=CH– group, supporting the presence of the unsaturated spacer. Increasing the length of the *N*-alkyl substituent does not lead to significant changes in the overall spectral profile, apart from the expected increase in the relative intensity of bands assigned to methylene groups, which reflects the increasing contribution of the hydrocarbon chains.

12-4ene-12 FTIR (ν_max_/cm^−1^): 2950, 2850, 1473, 1394, 960, 720;

14-4ene-14 FTIR (ν_max_/cm^−1^): 2950, 2848, 1472, 1373, 952, 719;

16-4ene-16 FTIR (ν_max_/cm^−1^): 2912, 2850, 1464, 1382, 959, 720;

18-4ene-18 FTIR (ν_max_/cm^−1^): 2999, 2830, 1490, 1380, 970, 730.

### 2.2. Micellization and Aggregation Properties of Gemini Surfactants

At the interface, molecules of gemini surfactants orient themselves in such a way that the hydrophilic quaternary ammonium groups remain in contact with the aqueous phase, whereas the hydrophobic alkyl chains are directed away from the polar environment. The progressive adsorption of surfactant molecules results in a decrease in surface tension until the interface becomes saturated and further addition of surfactant promotes the formation of supramolecular aggregates in the bulk phase. The concentration at which this process becomes significant is described by the critical micelle concentration (CMC). Owing to the cooperative action of two hydrophobic chains within a single molecule, gemini surfactants typically exhibit considerably lower CMC values than their monomeric analogues [38,39,40,41,42,43].

The aggregation properties of but-2-ene-1,4-bis(*N*-alkyl-*N*,*N*-dimethylammonium bromides) were evaluated by conductometric measurements. The CMC values were determined from the intersection of the linear regressions corresponding to the pre-micellar and micellar concentration ranges. The degree of counterion binding, β, was calculated as β = 1 − α, where α represents the ratio of the slopes above and below the CMC [36]. The standard Gibbs energy of micellization (ΔG°mic) values were calculated by the following equation:

ΔG°mic = 2RT(½ + β)lnCMC − RTln2

where R is the universal gas constant, T is the absolute temperature (298.15 K), β is the degree of counterion binding, and CMC is the critical micelle concentration expressed in mol L^−1^. The calculated ΔG°mic values are reported per mole of gemini surfactant.

The aggregation parameters of the investigated but-2-ene-1,4-bis(*N*-alkyl-*N*,*N*-dimethylammonium bromides) are summarized in Table 3. The pronounced decrease in CMC from C12 to C16 is consistent with the general behavior of homologous gemini surfactant series, in which increasing hydrocarbon-chain length strengthens the hydrophobic driving force for aggregation [44,45,46,47]. However, the increase observed for the C18 homologue indicates that, beyond a certain chain length, additional structural factors may become important. These may include less favorable molecular packing, reduced conformational adaptability, and changes in hydration of the interfacial region. Therefore, the aggregation behavior of the longest-chain derivative cannot be explained solely by hydrophobicity.

The counterion binding parameters also changed with alkyl-chain length, indicating differences in the packing and surface charge density of the micelles. The negative values of ΔG°mic obtained for all homologues confirm that micellization is thermodynamically spontaneous. The most negative value observed for the C16 derivative is consistent with its particularly low CMC and indicates that aggregation is most favorable for this homologue. The relatively high β values indicate substantial association of bromide counterions with the positively charged micellar surface. Variations in β across the homologous series may reflect differences in head-group separation, local charge density, and micellar packing. Such changes can influence both electrostatic repulsion between cationic head groups and the overall thermodynamic stability of the aggregates.

A comparison with the saturated analogue further indicates that the presence of the double bond in the spacer increases the tendency toward micellization. The 12-4-12 compound, containing a four-carbon spacer composed exclusively of single bonds, exhibits a CMC of 1.20 mM [48,49], whereas the corresponding unsaturated 12-4ene-12 derivative shows a lower CMC of 0.90 mM. This decrease suggests that spacer rigidification caused by the C=C double bond facilitates aggregation and increases the tendency of the molecules to form micelles. The effect may be related to changes in molecular conformation and packing induced by the unsaturated spacer. Thus, not only the length of the hydrophobic chains but also the structural rigidity of the spacer contributes to the aggregation behavior of these gemini surfactants.

### 2.3. Minimum Concentrations Inhibiting the Growth of Microorganisms

In the next stage of the study, the minimum concentrations of the tested compounds that inhibited the growth of both bacteria and microscopic fungi were determined. The results are presented in Table 4. All MIC measurements were performed under identical medium-composition and ionic-strength conditions for all tested reference strains.

The minimum inhibitory concentrations determined for all compounds range from 0.06 mM to 31.25 mM. Among the tested compounds, 12-4ene-12 exhibits the highest antibacterial activity (the lowest MIC values for all strains). The susceptibility ranking for this compound can be expressed as follows: *C. albicans* (yeast) < *S. aureus* (Gram-positive bacteria) = *E. coli* (Gram-negative bacteria) < *A. niger* (mold) < *P. chrysogenum* (mold). As the hydrophobicity of a molecule increases (by increasing the number of methylene groups in the chains), MIC values rise by as much as several orders of magnitude. In extreme cases, for *E. coli*, these values range from 0.12 mM to >62.5 mM (lower-bound result). This phenomenon may potentially stem from the so-called “cut-off effect,” which relates to changes in the physicochemical properties of compounds. Surfactants with longer chains have significantly lower CMC. It is possible that in solution, they exhibit a strong tendency to form large, stable aggregates, which drastically reduces the concentration of free, monomeric molecules. It is likely that monomeric molecules are capable of penetrating the cell walls and membranes of microorganisms, causing their destabilization. Furthermore, an excessively long hydrocarbon chain hinders transport across membranes, which limits the compound’s penetration into the lipid bilayer [50]. It should be noted, however, that the studies conducted did not analyze compounds with alkyl chains shorter than C12; therefore, this series of investigations does not allow for the determination of the global optimum chain length.

As the hydrophobic chain lengthens, a significant difference in susceptibility between Gram-negative and Gram-positive bacteria becomes apparent. For compound 12-4ene-12, both types of bacteria exhibit identical susceptibility at 0.12 mM. Starting with compound 14-4ene-14, *E. coli* becomes drastically less susceptible (the MIC rises sharply to 7.81 and >62.5 mM), while *S. aureus* remains relatively susceptible (MIC at 0.24; 1.95 and 15.62 mM). This phenomenon stems directly from fundamental differences in cell envelope structure. Gram-negative bacteria possess an asymmetric outer membrane rich in lipopolysaccharides (LPS), which acts as a highly effective hydrophobic barrier. High-molecular-weight or highly aggregated gemini surfactants with longer chains encounter steric and electrostatic resistance, which prevents them from reaching the cytoplasmic membrane. In contrast, the Gram-positive *S. aureus* has only a porous peptidoglycan layer, which does not constitute such a restrictive diffusion barrier for amphiphilic molecules [51,52,53].

An interesting aspect of the compounds studied is their high activity against microscopic fungi. Within the analyzed range of alkyl chain lengths (homologs from C12 to C16), the MIC values against *Candida albicans* and *Aspergillus niger* were characterized by a concentration range below 1 mM, ranging from 0.06 to 0.98 mM. Only with compound 18-4ene-18 was a reduction in antifungal activity observed. The high bioactivity of cationic gemini surfactants stems from their strong electrostatic affinity for the polar, negatively charged components found in the cell wall and cell membrane of microscopic fungi. This structure promotes the permeabilization of ergosterol-rich cell membranes, which consequently leads to destruction of some cells. Based on the literature [53,54,55], one could hypothesize that the higher MIC values recorded for *Penicillium chrysogenum* may stem from the specific composition of its cell wall. An increased content of glucans or chitin often serves as a significant diffusion barrier against antimicrobial compounds.

### 2.4. The Influence of the Tested Compounds on the Development of Escherichia coli

**Figure 4:**
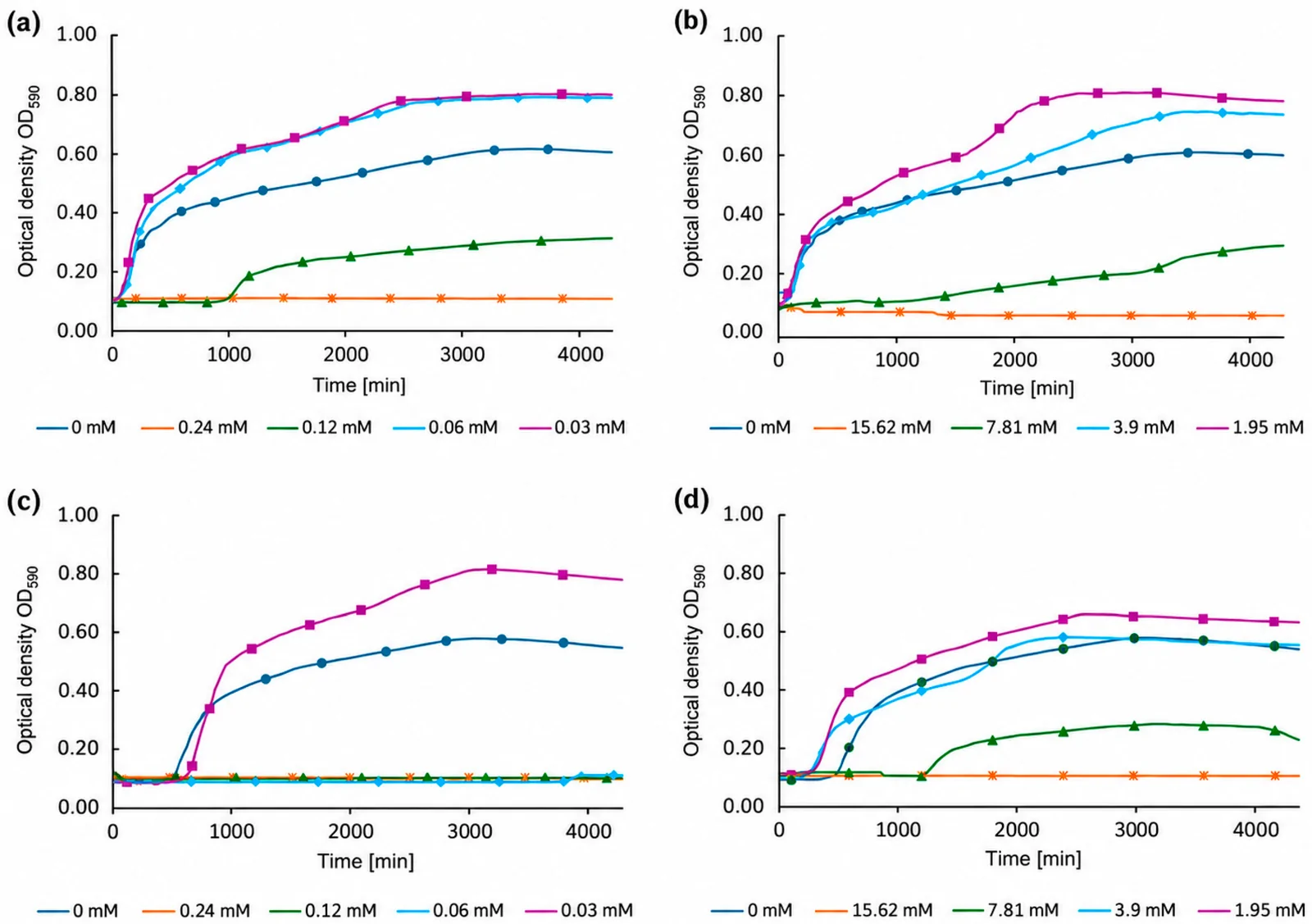
Growth of *Escherichia coli* after exposure to gemini surfactant 12-4ene-12 (**a**,**c**) and 14-4ene-14 (**b**,**d**) at the initial inoculum value 1.0 × 10^8^ CFU/mL (**a**,**b**) or 1.0 × 10^6^ CFU/mL (**c**,**d**).

As compounds 12-4ene-12 and 14-4ene-14 exhibited the highest antimicrobial activity, the next phase of testing evaluated their effect on changes in the biomass of *Escherichia coli* (Figure 4).

Gemini surfactants with dodecyl chains (12-4ene-12) did not inhibit the growth of *Escherichia coli* at the two lowest concentrations analyzed (0.03 and 0.06 mM) (Figure 4a). Microorganism proliferation, similar to the control sample, began without a distinct lag phase. However, an increase in final biomass was observed—the samples reached an OD590 of 0.8, which was significantly higher than that of the control. In samples where the concentration of 12-4ene-12 was 0.12 mM, effective inhibition of bacterial growth was observed during the first 1000 min of incubation. After this time, a slow, limited increase in biomass occurs, reaching a value of only OD590 = 0.31 at the final measurement point (4000 min). In contrast, the compound at the highest tested concentration of 0.24 mM causes complete suppression of *Escherichia coli* growth.

In the experimental setup using an inoculum with an initial density 2 log lower (Figure 4c), the adaptation phase in the sample without the gemini surfactant lasted until approximately 440 min. Subsequently, biomass growth was observed, and the maximum optical density (OD590 = 0.57) was recorded after approximately 50 h of cultivation.

In the presence of compound 12-4ene-12 at a concentration of 0.03 mM, the adaptation phase was prolonged to approximately 700 min, followed by a rapid exponential growth. Ultimately, the bacterial culture reached a higher final density (OD590 = 0.8) than the control. In samples where the compound concentration ranged from 0.06 to 0.25 mM, complete inhibition of bacterial growth was observed throughout the entire duration of the experiment (4300 min). The OD590 value remained at a constant, low level of 0.1. For *Escherichia coli* with an initial density of 1.0 × 10^6^ CFU/mL, a concentration of the compound 12-4ene-12 equal to 0.06 mM can be considered the MIC value.

When the gemini surfactant with longer alkyl chains (14-4ene-14) was used at the highest concentration tested (15.62 mM), complete inhibition of bacterial growth was observed throughout the entire duration of the experiment (Figure 4b). The curve remained stable, and the OD590 value did not exceed 0.12. The compound at half that concentration (7.81 mM) also demonstrated high bactericidal efficacy. By the end of the experiment (after 56 h), an increase in the OD590 value to 0.3 was observed. The first signs of bacterial growth were recorded after 1460 min (with an OD590 value of 0.15). In the samples containing the gemini surfactant at concentrations of 3.9 mM and 1.95 mM, the growth curve profiles were similar to those of the control sample (0 mM). The maximum OD590 values were 0.78 and 0.76, respectively, representing a biomass increase of more than 25 percentage points higher than in the samples which did not contain a biocide.

For cultures with an inoculum density of 1.0 × 10^6^ CFU/mL treated with the surfactant 14-4ene-14 at a concentration of 16 mM, no bacterial growth was observed throughout the entire duration of the experiment (Figure 4d). In samples containing the biocide at a concentration of 7.81 mM, a reduction in microbial growth was also observed. The initial incubation phase was characterized by low optical density values, but after approximately 1320 min, a moderate increase occurred. No growth-inhibitory effect was observed for samples at concentrations of 1.95 mM and 3.9 mM. At a concentration of 1.95 mM, the highest optical density values were obtained among all analyzed samples (OD590 = 0.65). A similar trend was observed for *E. coli* cultures containing the gemini surfactant at a concentration of 3.9 mM, where the final OD590 values were similar to those of the control sample. However, the observed increase in optical density does not necessarily reflect a proportional increase in biomass and may partly result from cell aggregation or surfactant-induced changes in cell morphology.

At the limit concentrations (0.12 mM for compound 12-4ene-12 and 7.81 mM for compound 14-4ene-14), a prolongation of the lag phase (up to 1000 min) or a secondary growth surge at the end of a long incubation period (after 56 h) is observed. It is likely that bacteria have the ability, over time, to adapt to sublethal doses of surfactant, metabolize it, or select for cells with lower sensitivity, leading to a resumption of proliferation. In samples with sub-MIC concentrations (0.03–0.06 mM for 12-4ene-12 and 2–4 mM for 14-4ene-14), no inhibition was observed, and the final biomass increase exceeded that of the control by more than 25%. If we accept this hypothesis, we can suppose that this effect may result from the phenomenon of hormesis: sublethal concentrations of the surfactant induce moderate protective stress, stimulating the biosynthesis of cell wall polysaccharides, the remodeling of membrane proteins, or increased cell aggregation, which optically inflates the absorbance measurement of the culture [53,56]. These hypotheses should be confirmed in further studies. At intermediate concentrations (0.12 mM for 12-4ene-12 and 7.81 mM for 14-4ene-14), the observed growth delay (lag phase up to 1000 min) indicates a temporary inhibition of cell division and a slow selection or adaptation of the bacterial population to the presence of the biocide [20,53].

Significant differences in growth rates between cultures with different initial densities (Figure 4a,b vs. Figure 4c,d) confirm that antibacterial activity is strongly dependent on inoculum size. Reducing the inoculum by two orders of magnitude (to 1.0 × 10^6^ CFU/mL) resulted in a concentration of 0.06 mM of the compound 12-4ene-12—which had been completely ineffective in a denser culture—leading to complete suppression of proliferation for 4300 min. The literature [35,52,56] suggests that a high bacterial population density leads to the distribution of the pool of free molecules among a large number of cells, preventing the attainment of a critical concentration on their surfaces—a concentration necessary to damage external structures. Determining whether this phenomenon also occurs in the system under study requires further, in-depth experimental analysis. Differences are also evident between the methods used to determine the inhibitory effects of gemini surfactants. The MIC value for compound 14-4ene-14, determined after 24 h for an *Escherichia coli* population in the order of 10^7^ in the first experiment, is 7.81 mM. In the second experiment, however—using the Odin BIOLOG system—biomass growth was observed after 24 h for both 10^6^ and 10^8^ inoculum sizes. It should be noted, however, that these determined concentration values represent the minimum concentrations required to inhibit microbial growth, rather than the concentrations at which the compound exhibits a biocidal effect. Determining such values always constitutes the first stage of research, clearly indicating which concentration ranges should be included in subsequent experimental phases concerning the antimicrobial activity of the compounds. Also, it is not possible to draw definitive conclusions regarding the mechanism of action of the compounds studied based on the data collected. The descriptions of the mechanisms are purely hypothetical and serve as a starting point for further studies.

### 2.5. Quantum Chemical Calculations

**Figure 5:**
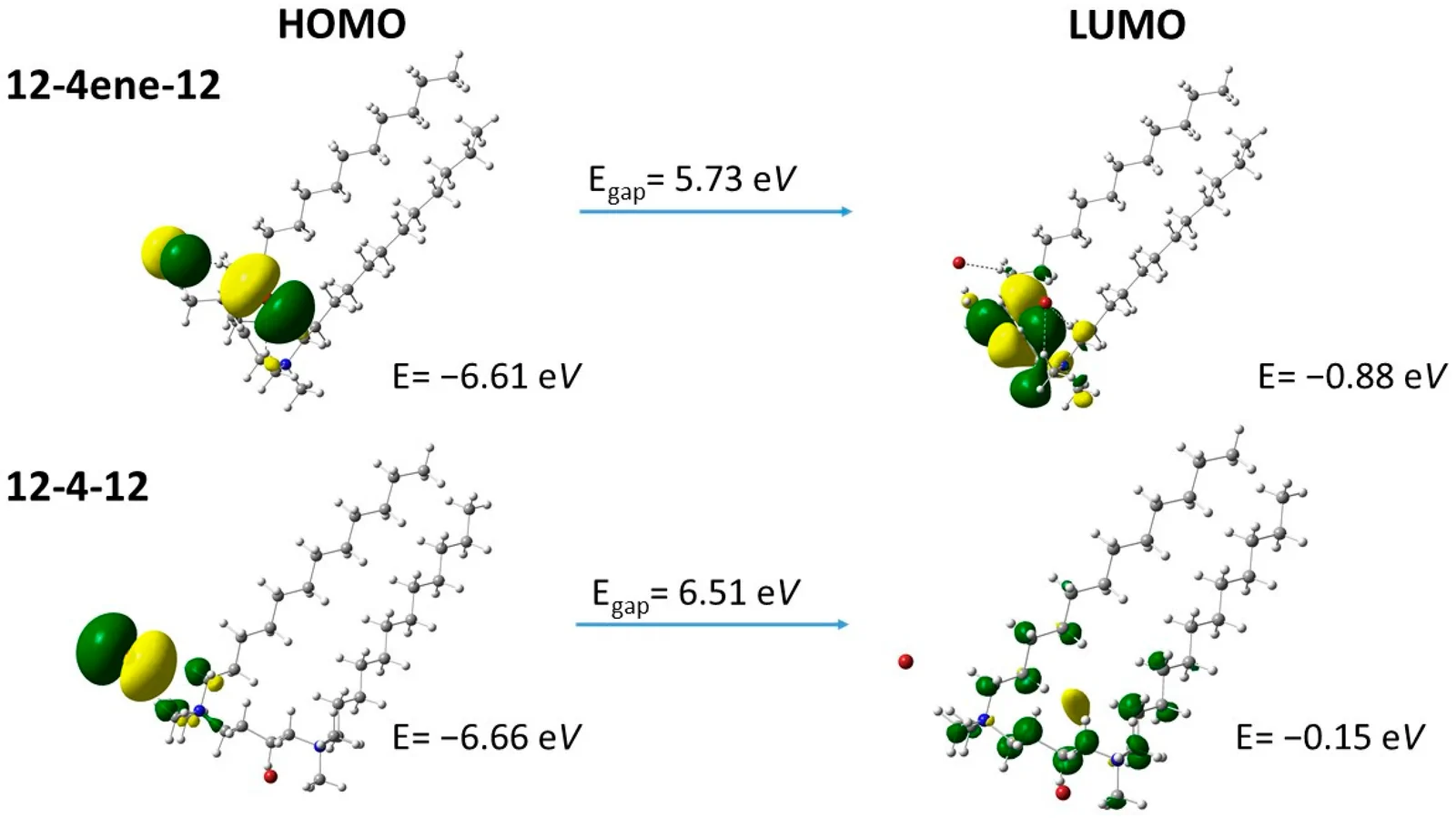
Visualization of the frontier molecular orbitals of compounds 12-4ene-12 and 12-4-12. The green and yellow isosurfaces correspond to opposite signs of the orbital wavefunction.

Calculations were performed for the 12-4ene-12 and 12-4-12 molecules, which differ in the structure of the spacer. The aim of the study was to evaluate the effect of spacer rigidification resulting from the presence of a double bond on the reactivity of the surfactant. A saturated analogue was selected as a reference compound to allow for a direct assessment of the impact of unsaturation in the linker, while minimizing other structural differences. To account for interactions with the surrounding environment, the calculations were carried out using a solvent model representing an aqueous medium. As key descriptors of the electronic properties and reactivity of the investigated compounds, the energies and distributions of the frontier molecular orbitals (HOMO and LUMO) were analyzed. In both analyzed systems, the HOMO is mainly localized in the vicinity of the bromide anion, indicating this region as a potential electron-donating site. In contrast, the LUMO distribution is strongly influenced by the structure of the spacer. For 12-4-12, the LUMO is delocalized over a significant part of the spacer fragment and in the vicinity of the quaternary nitrogen atoms. In the case of 12-4ene-12, the LUMO is primarily localized within the spacer fragment containing the C=C double bond (Figure 5).

**Figure 6:**
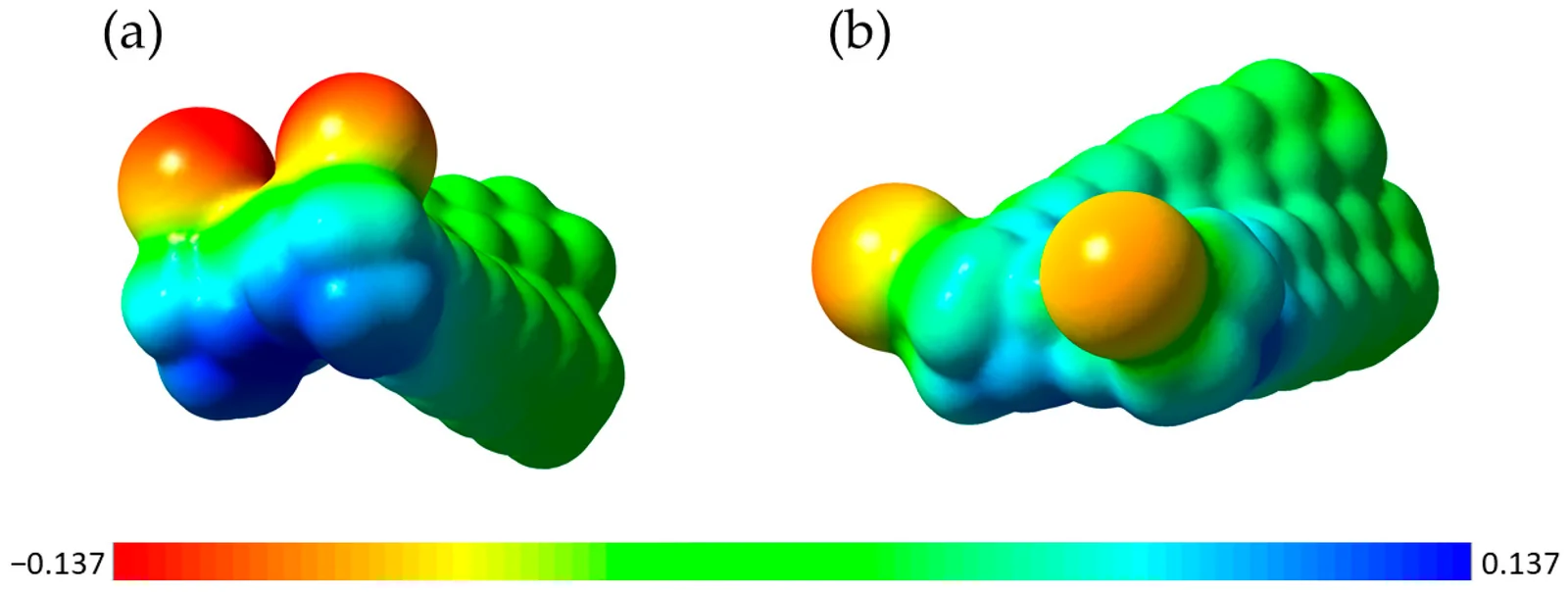
Electrostatic potential of (**a**) 12-4ene-12 and (**b**) 12-4-12 mapped on an electron density isosurface. The blue color is for the positive potential and the red color is for the negative one. The electrostatic potential was mapped onto an electron-density isosurface of 0.0004 a.u., and the electrostatic potential scale is expressed in atomic units, with values ranging from −0.137 to 0.137.

The HOMO–LUMO energy gap provides insight into the electronic properties and potential charge-transfer behavior of the investigated compounds (Table 5). A lower Egap is generally associated with greater electronic softness and an increased propensity for electron redistribution. Accordingly, the smaller Egap value of 12-4ene-12 (5.73 eV), compared with that of 12-4-12 (6.51 eV), suggests greater electronic softness and a higher tendency toward charge transfer. Nevertheless, these parameters alone are insufficient to draw conclusions regarding antimicrobial activity, which is a multifactorial property determined by numerous physicochemical and biological factors; therefore, it cannot be reliably predicted solely from the HOMO-LUMO gap. Electrostatic potential maps (MEP) depict the spatial distribution of electrostatic potential over the molecular surface, enabling the identification of electron-rich and electron-deficient regions and providing insight into the ability of a molecule to participate in electrostatic interactions and form noncovalent bonds [57,58,59]. In the case of antimicrobial compounds, the presence of regions characterized by a positive electrostatic potential may facilitate interactions with negatively charged components of bacterial cell surfaces, which is considered one of the factors influencing their biological activity. Electrostatic potential maps were generated for compounds 12-4ene-12 and 12-4-12 (Figure 6).

Analysis of the obtained surfaces reveals a clear separation between electron-rich and electron-deficient regions. Areas exhibiting the most negative electrostatic potential (red) are associated with the presence of Br^−^ counterions, whereas the positive electrostatic potential (blue) is concentrated around the quaternary nitrogen atoms. Regions with electrostatic potential values close to zero (green) are observed on the hydrophobic alkyl chains. Compared with 12-4-12, the 12-4ene-12 molecule exhibits higher positive electrostatic potential values and a larger difference between the extreme positive and negative potential values. This indicates a more pronounced charge polarization on the surface of the surfactant containing the C=C double bond. The increased polarization may enhance electrostatic interactions with negatively charged components of bacterial cell surfaces, potentially strengthening the ability of the compound to interact with microbial cell membranes.

## 3. Materials and Methods

### 3.1. Materials

*N*-Dodecyl-*N*,*N*-dimethylamine (97%), *N*-tetradecyl-*N*,*N*-dimethylamine (≥95%), *N*-hexadecyl-*N*,*N*-dimethylamine (≥95%), and trans-1,4-dibromobut-2-ene (99%) were purchased from Merck (Poznan, Poland). *N*-Octadecyl-*N,N*-dimethylamine (>90%) was obtained from TCI (Tokyo, Japan). Acetonitrile (≥99%), methanol (≥99%), and phosphorus pentoxide (98%) were purchased from VWR Chemicals (Gdansk, Poland). All reagents and solvents were used as received without further purification.

### 3.2. Synthesis of Gemini Surfactants

The series of but-2-ene-1,4-bis(*N*-alkyl-*N*,*N*-dimethylammonium bromides) was synthesized by quaternization of 1,4-dibromobut-2-ene with the corresponding *N*-alkyl-*N*,*N*-dimethylamines. The reactions were carried out using a 1:2 molar ratio of 1,4-dibromobut-2-ene to tertiary amine in acetonitrile under reflux conditions. The reaction mixtures were heated until quaternization was complete, which was accompanied by the progressive formation of a solid product. The crude products were isolated and recrystallized from an acetonitrile/methanol mixture (10:1, *v*/*v*), followed by drying over phosphorus pentoxide. The synthesized compounds were obtained in high yields.

### 3.3. Analytical Methods

Melting points were measured using a Stuart SMP30 apparatus (Staffordshire, UK) in capillary tubes sealed at one end. The 1H and 13C NMR spectra were recorded using a Varian VNMR-S spectrometer (Oxford, UK), operating at 403 MHz for 1H and 101 MHz for 13C. Measurements were carried out in CDCl_3_ with tetramethylsilane (TMS) as the internal reference. Chemical shifts are reported as δ values in ppm. FTIR spectra were recorded using a Bruker IFS 66v/S spectrometer (Poznan, Poland). Samples were analyzed in the solid state using potassium bromide pellets. The complete NMR and FTIR datasets are available from the corresponding author upon reasonable request.

### 3.4. Conductivity Measurements

The critical micelle concentrations of the synthesized surfactants were determined by conductivity measurements using an Elmetron CC-505 conductivity meter (Zabrze, Poland). Prior to analysis, the instrument was calibrated with a conductivity standard of 147 µS cm^−1^ at 298.15 K. All solutions were prepared with double-distilled water and measured at 298.15 K. Particular attention was paid to the longer-chain homologues, 16-4ene-16 and 18-4ene-18, and measurements were performed only after complete dissolution and formation of homogeneous solutions. CMC values were obtained from conductivity versus concentration plots. Two linear regions were identified, corresponding to the pre-micellar and micellar concentration ranges, and the CMC was taken as the concentration at the intersection of the two regression lines. Each determination was repeated at least three times, and the mean value was used for further analysis.

### 3.5. Antimicrobial Activity

Gemini compounds were tested for antimicrobial activity against bacteria (*Escherichia coli* ATCC 10536, *Staphylococcus aureus* ATCC 6538) and microscopic fungi (*Candida albicans* ATCC 10231, *Aspergillus niger* ATCC 16401, and *Penicillium chrysogenum* ATCC 60739). The MIC values for microorganisms were determined by a tube standard two-fold dilution method [29]. Each microorganism was resuspended in physiological saline solution (molds in water with Tween 80 0.1% addition) and diluted to 2 × 10^7^ CFU/mL for bacteria and 2 × 10^6^ CFU/mL for fungi. In the next step, 1 mL of microorganism suspension was mixed with 1 mL of media (TSB-Tryptic Soy Broth (MERCK, Darmstadt, Germany) for bacteria, and MEB Malt Extract Broth (MERCK Darmstadt, Germany) for microscopic fungi) containing serial dilutions of the tested compounds. The final concentration of microorganisms in the samples was 1 × 10^7^ CFU/mL for bacteria and 1 × 10^6^ CFU/mL for fungi. The concentration range of the gemini surfactants studied, in turn, was between 62.5 and 0.0038 mM. Bacteria and yeast samples were incubated at 37 °C (bacteria for 24 h, yeast for 48 h), and the molds were incubated at 28 °C for 48 h. As a growth control, a suspension of microorganisms in a medium without the biocides was used. The studies included also sterility testing and blank tests using a medium containing a surfactant but without microorganisms. The MICs were defined as the lowest concentration of the compounds in which there was no visible growth. All tests were repeated three times.

### 3.6. The Influence of the Tested Compounds on the Development of Escherichia coli

To determine changes in bacterial biomass over time in the presence of the gemini surfactants 12-4ene-12 or 14-4ene-14, the BIOLOG Odin system (Biolog, Inc.; Hayward, CA, USA) was used. Samples prepared in the same manner as described in Section 3.5. were dispensed into 96-well plates at a volume of 200 µL per well. Control samples consisted of a medium containing an inoculum without biocidal compounds. All tests were performed in triplicate. The studies utilized concentrations of gemini surfactants both above and below the MIC values, ranging from 0.03–0.24 mM for compound 12-4ene-12 and 1.95–15.62 mM for 14-4ene-14. At this stage of the work, inocula of two different densities—2 × 10^8^ and 2 × 10^6^ CFU/mL—were used. The final concentrations of *Eschericia coli* in the samples were 1 × 10^8^ and 1 × 10^6^ CFU/mL The studies were conducted on *Escherichia coli* ATCC 10536. The prepared plates were placed in an Odin BIOLOG apparatus (Biolog, Inc.; Hayward, CA, USA), and the change in the optical density of the microorganisms was measured over a 72-h period.

### 3.7. Computational Quantum Mechanical Modeling Method

DFT calculations were performed using the GAUSSIAN16 program package [60]. The APF-D functional [61,62] and the 6-311++G(d,p) [63] basis set were selected because they provide an optimal balance between computational efficiency and the accuracy of the calculated results. The molecular charge and spin multiplicity were set to 0 and 1, respectively. The optimization procedure was carried out in two steps. First, with the alkyl chains kept fixed, a variety of initial positions of the bromide anion were considered, and the lowest-energy structures were selected. Subsequently, full geometry optimizations were performed starting from different conformations of the alkyl chains. As several of the investigated structures did not reach convergence, the lowest-energy structures among the successfully converged geometries were selected for further analysis. All calculations were performed in an aqueous environment, accounting for solvent effects using the Polarizable Continuum Model (PCM) [64]. In this approach, the molecule is placed inside a cavity embedded in a dielectric continuum representing water with a static dielectric constant of 78.3553 and an optical dielectric constant of 1.777849, reproducing the bulk polarization properties of liquid water. The cavity is constructed from scaled van der Waals spheres using UFF (Universal Force Field) atomic radii, enlarged by a scaling factor of 1.10. This setup models aqueous solvation efficiently while avoiding the computational cost of including explicit water molecules. The HOMO and LUMO energy values, initially obtained in atomic units (a.u.), were converted to electronvolts (eV) according to the relation 1 a.u. = 27.2114 eV. The optimized geometries of studied molecules are available from the corresponding author upon request.

## 4. Conclusions

A homologous series of unsaturated quaternary ammonium gemini surfactants containing a semi-rigid but-2-ene spacer and C12–C18 alkyl chains was successfully synthesized and characterized. Conductometric measurements revealed that the critical micelle concentration decreased with increasing alkyl-chain length from C12 to C16, reaching its lowest value of 0.026 mM for 16-4ene-16, followed by an increase for the C18 homologue. Comparison with the saturated analogue 12-4-12 showed that introducing a C=C double bond into the spacer reduced the CMC from 1.20 to 0.90 mM, suggesting that spacer rigidification favors micelle formation. Antimicrobial activity decreased markedly with increasing alkyl-chain length. The 12-4ene-12 derivative exhibited the lowest MIC values against all tested microorganisms, with particularly high activity against *Candida albicans* (0.06 mM) and both *Escherichia coli* and *Staphylococcus aureus* (0.12 mM). Longer-chain homologues showed substantially reduced activity, particularly against *E. coli*, demonstrating a pronounced cut-off effect. Growth-kinetic studies further confirmed the concentration- and inoculum-dependent inhibitory activity of 12-4ene-12 and 14-4ene-14 against *E. coli*. A limitation of the present study is that the antimicrobial experiments were performed using planktonic cultures and did not include biofilm models. Quantum chemical calculations revealed that the unsaturated spacer alters the electronic properties of the surfactants, resulting in a lower HOMO–LUMO energy gap and greater electrophilicity for 12-4ene-12 compared with its saturated analogue. Overall, the results highlight the distinct effects of alkyl-chain length and spacer structure on micellization and antimicrobial activity, providing insights into the structure–property relationships of gemini surfactants.

## Figures and Tables

**Table 1:** Proton chemical shifts from ^1^H NMR spectra of gemini surfactants (n-4ene-n) molecules.

Gemini	-CH=CH-	-N+CH_2_CH=	-N+CH_2_-	-N+(CH_3_)_2_	-N+CH_2_CH_2_-	-CH_2_(CH_2_)_x_CH_3_	-CH_2_CH_3_
12-4ene-12	7.14 (2H)	4.59 (4H)	3.58 (4H)	3.37 (12H)	1.73 (4H)	1.36–1.25 (36H)	0.88 (6H)
14-4ene-14	7.10 (2H)	4.60 (4H)	3.59 (4H)	3.37 (12H)	1.73 (4H)	1.35–1.25 (44H)	0.88 (6H)
16-4ene-16	7.10 (2H)	4.58 (4H)	3.57 (4H)	3.36 (12H)	1.78 (4H)	1.36–1.25 (52H)	0.88 (6H)
18-4ene-18	7.11 (2H)	4.59 (4H)	3.57 (4H)	3.36 (12H)	1.78 (4H)	1.35–1.26 (60H)	0.88 (6H)

**Table 2:** Carbon chemical shifts from ^13^C NMR spectra of gemini surfactants (n-4ene-n) molecules.

Gemini	-CH=CH-	-N+CH_2_CH=	-N+CH_2_-	-N+CH_3_	-N+CH_2_(CH_2_)_x_-	-CH_2_CH_3_	-CH_2_CH_3_
12-4ene-12	130.2	65.6	64.7	50.5	31.8–26.2	22.9	14.0
14-4ene-14	130.2	65.5	64.5	50.5	31.8–26.2	22.5	14.0
16-4ene-16	130.2	65.5	64.7	50.5	31.8–26.2	22.6	14.0
18-4ene-18	130.0	65.5	64.5	50.5	31.9–26.8	22.7	14.1

**Table 3:** Aggregation parameters for but-2-ene-1,4-bis(*N*-alkyl-*N*,*N*-dimethylammonium bromides).

Gemini Surfactants	CMC [mM]	β	ΔG°mic [kJ/mol]
12-4ene-12	0.90 ± 0.05	0.70	−43.4
14-4ene-14	0.42 ± 0.02	0.58	−43.3
16-4ene-16	0.026 ± 0.001	0.45	−51.5
18-4ene-18	0.14 ± 0.01	0.56	−48.3

**Table 4:** Minimum inhibitory concentrations (MIC [mM]) of but-2-ene-1,4-bis(*N*-alkyl-*N*,*N*-dimethylammonium bromides).

Gemini Surfactants	Microorganisms				
	*E. coli*	*S. aureus*	*C. albicans*	*A. niger*	*P. chrysogenum*
12-4ene-12	0.12	0.12	0.06	0.24	0.49
14-4ene-14	7.81	0.24	0.24	0.49	0.98
16-4ene-16	31.25	1.95	0.49	0.98	3.9
18-4ene-18	>62.5 *	15.62	1.95	3.9	7.81

* Lower-bound result.

**Table 5:** Frontier molecular orbital energies and global reactivity descriptors (in eV) of 12-4ene-12 and 12-4-12.

	12-4ene-12	12-4-12
E*HOMO* [eV]	−6.61	−6.66
E*LUMO* [eV]	−0.88	−0.15
E*gap* [eV]	5.73	6.51
µ [eV]	−3.75	−3.41
η [eV]	2.87	3.26
σ [eV^−1^]	0.17	0.15
ω [eV]	2.45	1.78
ΔNmax	1.31	1.05

µ—electronic chemical potential [µ = ELUMO + EHOMO)/2]; η—global hardness [η = (ELUMO − EHOMO)/2]; σ—global softness (σ = 1/(2η)); ω—global electrophilicity index (ω = μ2/2η); ΔNmax—maximum charge transfer index (ΔNmax = −μ/η).

## Data Availability

The data supporting the findings of this study are available in Supporting Information or from the corresponding author upon reasonable request.

## Supplementary Materials

The following supporting information can be downloaded at: https://www.mdpi.com/article/10.3390/molecules31213969/s1. Detailed synthetic procedures for compounds 12-4ene-12, 14-4ene-14, 16-4ene-16, and 18-4ene-18, together with their ^1^H and ^13^C NMR spectra (Figures S1–S8) and conductivity plot used for CMC determination (Figures S9–S12).

## References

[1] Menger F.M., Littau C.A. (1991). Gemini-Surfactants: Synthesis and Properties. J. Am. Chem. Soc..

[2] Menger F.M., Keiper J.S. (2000). Gemini Surfactants. Angew. Chem. Int. Ed..

[3] Menger F.M., Littau C.A. (1993). Gemini Surfactants: A New Class of Self-Assembling Molecules. J. Am. Chem. Soc..

[4] Zana R. (2002). Alkanediyl-α,ω-Bis(Dimethylalkylammonium Bromide) Surfactants. 10. Behavior in Aqueous Solution at Concentrations below the Critical Micellization Concentration: An Electrical Conductivity Study. J. Colloid Interface Sci..

[5] Brycki B.E., Kowalczyk I.H., Szulc A., Kaczerewska O., Pakiet M., Najjar R. (2017). Multifunctional Gemini Surfactants: Structure, Synthesis, Properties and Applications. Application and Characterization of Surfactants.

[6] Sharma R., Kamal A., Abdinejad M., Mahajan R.K., Kraatz H.-B. (2017). Advances in the Synthesis, Molecular Architectures and Potential Applications of Gemini Surfactants. Adv. Colloid Interface Sci..

[7] Kowalczyk I., Szulc A., Brycki B. (2025). Gemini Surfactants: Advances in Applications and Prospects for the Future. Molecules.

[8] Fatma N., Panda M., Ansari W.H. (2015). Kabir-ud-Din Solubility Enhancement of Anthracene and Pyrene in the Mixtures of a Cleavable Cationic Gemini Surfactant with Conventional Surfactants of Different Polarities. Colloids Surf. Physicochem. Eng. Asp..

[9] Zhou C., Wang F., Chen H., Li M., Qiao F., Liu Z., Hou Y., Wu C., Fan Y., Liu L. (2016). Selective Antimicrobial Activities and Action Mechanism of Micelles Self-Assembled by Cationic Oligomeric Surfactants. ACS Appl. Mater. Interfaces.

[10] Ahmady A.R., Hosseinzadeh P., Solouk A., Akbari S., Szulc A.M., Brycki B.E. (2022). Cationic Gemini Surfactant Properties, Its Potential as a Promising Bioapplication Candidate, and Strategies for Improving Its Biocompatibility: A Review. Adv. Colloid Interface Sci..

[11] Devinsky F., Lacko I., Mlynarčík D., Racansky V., Krasnec L. (1985). Relationship between Critical Micelle Concentration and Minimum Inhibitory Concentration for Some Non-Aromatic Quaternary Ammonium Salts and Amine Oxides. Tenside Surfactants Deterg..

[12] Islam M.S., Shortall S.M., Mekhail G.M., Callender S.P., Madkhali O., Bharwani Z., Ayyash D., Kobernyk K., Wettig S.D. (2017). Effect of Counterions on the Micellization and Monolayer Behaviour of Cationic Gemini Surfactants. Phys. Chem. Chem. Phys..

[13] Mirgorodskaya A.B., Kudryavtseva L.A., Pankratov V.A., Lukashenko S.S., Rizvanova L.Z., Konovalov A.I. (2006). Geminal Alkylammonium Surfactants: Aggregation Properties and Catalytic Activity. Russ. J. Gen. Chem..

[14] Pisárčik M., Polakovičová M., Markuliak M., Lukáč M., Devinsky F. (2019). Self-Assembly Properties of Cationic Gemini Surfactants with Biodegradable Groups in the Spacer. Molecules.

[15] Brycki B., Szulc A., Koenig H., Kowalczyk I., Pospieszny T., Górka S. (2019). Effect of the Alkyl Chain Length on Micelle Formation for Bis(N-Alkyl-N,N-Dimethylethylammonium)Ether Dibromides. Comptes Rendus Chim..

[16] Zana R. (1996). Critical Micellization Concentration of Surfactants in Aqueous Solution and Free Energy of Micellization. Langmuir.

[17] Pal N., Saxena N., Mandal A. (2017). Synthesis, Characterization, and Physicochemical Properties of a Series of Quaternary Gemini Surfactants with Different Spacer Lengths. Colloid Polym. Sci..

[18] Mivehi L., Bordes R., Holmberg K. (2011). Adsorption of Cationic Gemini Surfactants at Solid Surfaces Studied by QCM-D and SPR: Effect of the Rigidity of the Spacer. Langmuir.

[19] Li Q., Wang X., Zhuang W., Yao M., Pan Y., Chen X. (2021). Spacer Length Effect on the Aggregation Behaviours of Gemini Surfactants in EAN. Colloid Polym. Sci..

[20] Zhang S., Ding S., Yu J., Chen X., Lei Q., Fang W. (2015). Antibacterial Activity, *in Vitro* Cytotoxicity, and Cell Cycle Arrest of Gemini Quaternary Ammonium Surfactants. Langmuir.

[21] Xu Y., Gao H., Shen Y., Chai S., Zhang J., Zou Q. (2017). Application of Gemini Quaternary Ammonium with Ester Groups in Cationic P(St-Co-BA) Nanolatex and Study on Its Antibacterial Properties. Mater. Sci. Eng. C.

[22] Brycki B., Szulc A., Brycka J., Kowalczyk I. (2023). Properties and Applications of Quaternary Ammonium Gemini Surfactant 12-6-12: An Overview. Molecules.

[23] Minbiole K.P.C., Jennings M.C., Ator L.E., Black J.W., Grenier M.C., LaDow J.E., Caran K.L., Seifert K., Wuest W.M. (2016). From Antimicrobial Activity to Mechanism of Resistance: The Multifaceted Role of Simple Quaternary Ammonium Compounds in Bacterial Eradication. Tetrahedron.

[24] Zhao X., Li Y., Yuan H., Yin J., Hu M. (2017). Antibacterial Mechanism of Octamethylene-1,8-Bis(Dodecyldimethylammonium Bromide) Against *E. coli*. J. Surfactants Deterg..

[25] Devínsky F., Kopecka-Leitmanová A., Šeršeň F., Balgavý P. (1990). Cut-off Effect in Antimicrobial Activity and in Membrane Perturbation Efficiency of the Homologous Series of N,N-Dimethylalkylamine Oxides. J. Pharm. Pharmacol..

[26] Balgavý P., Devinsky F. (1996). Cut-off Effects in Biological Activities of Surfactants. Adv. Colloid Interface Sci..

[27] Koziróg A., Brycki B. (2015). Monomeric and Gemini Surfactants as Antimicrobial Agents—Influence on Environmental and Reference Strains. Acta Biochim. Pol..

[28] Laatiris A., El Achouri M., Rosa Infante M., Bensouda Y. (2008). Antibacterial Activity, Structure and CMC Relationships of Alkanediyl α,ω-Bis(Dimethylammonium Bromide) Surfactants. Microbiol. Res..

[29] Brycki B., Kowalczyk I., Kozirog A. (2011). Synthesis, Molecular Structure, Spectral Properties and Antifungal Activity of Polymethylene-α,ω-Bis(N,N-Dimethyl-N-Dodecyloammonium Bromides). Molecules.

[30] Obłąk E., Piecuch A., Krasowska A., Łuczyński J. (2013). Antifungal Activity of Gemini Quaternary Ammonium Salts. Microbiol. Res..

[31] Obłąk E., Piecuch A., Rewak-Soroczyńska J., Paluch E. (2019). Activity of Gemini Quaternary Ammonium Salts against Microorganisms. Appl. Microbiol. Biotechnol..

[32] Yang W., Cao Y., Wang Y., Ju H., Jiang Y., Geng T. (2021). Effects of Unsaturated Double Bonds on Adsorption and Aggregation Behaviors of Amide-Based Cationic Gemini Surfactants. Colloids Surf. Physicochem. Eng. Asp..

[33] Brycki B., Szulc A., Brycka J., Kowalczyk I. (2025). Cationic Gemini Surfactants in the Oil Industry: Applications in Extraction, Transportation and Refinery Products. Molecules.

[34] Tatsumi T., Zhang W., Nakatsuji Y., Miyake K., Matsushima K., Tanaka M., Furuta T., Ikeda I. (2001). Preparation, Surface-Active Properties, and Antimicrobial Activities of Bis(Alkylammonium) Dichlorides Having a Butenylen or a Butynylene Spacer. J. Surfactants Deterg..

[35] Fogang L.T., Kamal M.S., Hussain S.M.S., Kalam S., Patil S. (2020). Oil/Water Interfacial Tension in the Presence of Novel Polyoxyethylene Cationic Gemini Surfactants: Impact of Spacer Length, Unsaturation, and Aromaticity. Energy Fuels.

[36] Brycki B.E., Szulc A., Kowalczyk I., Koziróg A., Sobolewska E. (2021). Antimicrobial Activity of Gemini Surfactants with Ether Group in the Spacer Part. Molecules.

[37] Brycki B., Szulc A., Babkova M. (2020). Synthesis of Silver Nanoparticles with Gemini Surfactants as Efficient Capping and Stabilizing Agents. Appl. Sci..

[38] Jiang N., Li P., Wang Y., Wang J., Yan H., Thomas R.K. (2004). Micellization of Cationic Gemini Surfactants with Various Counterions and Their Interaction with DNA in Aqueous Solution. J. Phys. Chem. B.

[39] Pisárčik M., Jampílek J., Devínsky F., Drábiková J., Tkacz J., Opravil T. (2016). Gemini Surfactants with Polymethylene Spacer: Supramolecular Structures at Solid Surface and Aggregation in Aqueous Solution. J. Surfactants Deterg..

[40] Bhadani A., Endo T., Sakai K., Sakai H., Abe M. (2014). Synthesis and Dilute Aqueous Solution Properties of Ester Functionalized Cationic Gemini Surfactants Having Different Ethylene Oxide Units as Spacer. Colloid Polym. Sci..

[41] Chang H., Cui Y., Wang Y., Li G., Gao W., Li X., Zhao X., Wei W. (2016). Wettability and Adsorption of PTFE and Paraffin Surfaces by Aqueous Solutions of Biquaternary Ammonium Salt Gemini Surfactants with Hydroxyl. Colloids Surf. A.

[42] Aiad I., Khalil E., Farag A.E., Ahmed A., Abd-Elaal A. (2021). Ester-Gemini and Monomeric Cationic Surfactants; Synthesis, Characterization, Surface Parameters and Biological Activity. Egypt. J. Chem..

[43] Tikariha D., Ghosh K.K., Quagliotto P., Ghosh S. (2010). Mixed Micellization Properties of Cationic Monomeric and Gemini Surfactants. J. Chem. Eng. Data.

[44] Li Y., Li P., Wang J., Wang Y., Yan H., Thomas R.K. (2005). Odd/Even Effect in the Chain Length on the Enthalpy of Micellization of Gemini Surfactants in Aqueous Solution. Langmuir.

[45] Ansari W.H., Aslam J., Siddiqui U.S. (2012). Kabir-ud-Din Micellar Growth of M-2-m Type Gemini Surfactants (M=10, 12, 14) with Higher Chain Length Alcohols/Amines (C6–C8) in the Absence and Presence of Organic Salts (Sodium Salicylate, Sodium Tosylate). J. Mol. Liq..

[46] Hajy Alimohammadi M., Javadian S., Gharibi H., Tehrani-Bagha A.R., Alavijeh M.R., Kakaei K. (2012). Aggregation Behavior and Intermicellar Interactions of Cationic Gemini Surfactants: Effects of Alkyl Chain, Spacer Lengths and Temperature. J. Chem. Thermodyn..

[47] Oda R., Candau S.J., Huc I. (1997). Gemini Surfactants, the Effect of Hydrophobic Chain Length and Dissymmetry. Chem. Commun..

[48] Kuperkar K., Modi J., Patel K. (2012). Surface-Active Properties and Antimicrobial Study of Conventional Cationic and Synthesized Symmetrical Gemini Surfactants. J. Surfactants Deterg..

[49] Mozrzymas A. (2013). Modelling of the Critical Micelle Concentration of Cationic Gemini Surfactants Using Molecular Connectivity Indices. J. Solut. Chem..

[50] Pisárčik M., Bajcura M., Lukáč M., Devínsky F., Bilková A., Bilka F., Horváth B. (2023). Self-Aggregation of Cationic Gemini Surfactants with Amide Groups in the Spacer and Variable Alkyl Chain Length. Colloid Polym. Sci..

[51] Liang Y., Li H., Ji J., Wang J., Ji Y. (2023). Self-Aggregation, Antimicrobial Activity and Cytotoxicity of Ester-Bonded Gemini Quaternary Ammonium Salts: The Role of the Spacer. Molecules.

[52] Mechken K.A., Menouar M., Talbi Z., Saidi-Besbes S., Belkhodja M. (2024). Self-Assembly and Antimicrobial Activity of Cationic Gemini Surfactants Containing Triazole Moieties. RSC Adv..

[53] Tripathi V., Bhadra J., Bhattacharya S. (2026). Structure–Activity Relationships and Practical Aspects of Cationic Gemini Surfactants as Antimicrobial Agents. J. Surfactants Deterg..

[54] Fait M.E., Bakas L., Garrote G.L., Morcelle S.R., Saparrat M.C.N. (2019). Cationic Surfactants as Antifungal Agents. Appl. Microbiol. Biotechnol..

[55] Wegner T., Elias R., Roling L., Raj N., Gerke V., Fridman M., Glorius F. (2022). Cationic, Steroid-Based Imidazolium Amphiphiles Show Tunable Backbone-Dependent Membrane Selectivity in Fungi. ACS Infect. Dis..

[56] Rzycki M., Kaczorowska A., Kraszewski S., Drabik D. (2021). A Systematic Approach: Molecular Dynamics Study and Parametrisation of Gemini Type Cationic Surfactants. Int. J. Mol. Sci..

[57] Politzer P., Laurence P.R., Jayasuriya K. (1985). Molecular Electrostatic Potentials: An Effective Tool for the Elucidation of Biochemical Phenomena. Environ. Health Perspect..

[58] Varadwaj P.R., Marques H.M., Varadwaj A., Grabowski I., Yamashita K. (2026). Molecular Electrostatic Surface Potential: A Predictive Framework for Noncovalent Interactions and Adsorption Characteristics in Molecular Entities. Int. J. Mol. Sci..

[59] Gadre S.R., Suresh C.H., Mohan N. (2021). Electrostatic Potential Topology for Probing Molecular Structure, Bonding and Reactivity. Molecules.

[60] Frisch M.J., Trucks G.W., Schlegel H.B., Scuseria G.E., Robb M.A., Cheeseman J.R., Scalmani G., Barone V., Petersson G.A., Nakatsuji H. (2016). Gaussian 16.

[61] Austin A., Petersson G.A., Frisch M.J., Dobek F.J., Scalmani G., Throssell K. (2012). A Density Functional with Spherical Atom Dispersion Terms. J. Chem. Theory Comput..

[62] Foresman J.B., Frisch A. (2015). Exploring Chemistry with Electronic Structure Methods.

[63] Hehre W.J., Radom L., Schleyer P.V.R., Pople J.A. (1986). Ab Initio Molecular Orbital Theory.

[64] Tomasi J., Mennucci B., Cammi R. (2005). Quantum Mechanical Continuum Solvation Models. Chem. Rev..

