# Supplementary material for: Unsaturated Gemini Surfactants with a Semi-Rigid Spacer: Micellization and Antimicrobial Activity
Source: Molecules. 2026 Nov 3;31(21):3969. doi: 10.3390/molecules31213969 (PMC13765120; doi:10.3390/molecules31213969)
Supplement: Supplementary file 1 [file molecules-31-03969-s001.zip › molecules-4626780-supplementary.pdf]

Supporting Information

Unsaturated Gemini Surfactants with a Semi-Rigid Spacer: Micellization and Antimicrobial Activity

Iwona Kowalczyk, Adrianna Szulc, Justyna Brycka, Anna Komasa, Anna Koziróg, Bartosz Grabowski and Bogumił Brycki*

* Corresponding author: brycki@amu.edu.pl

Contents

- S1. General synthetic procedure
- S2. Detailed synthesis of individual gemini surfactants
- S3. NMR spectra
- S4. Conductivity plots used for CMC determination

S1. General synthetic procedure

A homologous series of but-2-ene-1,4-bis(*N*-alkyl-*N,N*-dimethylammonium bromides) was prepared by a Menshutkin quaternization reaction. trans-1,4-Dibromobut-2-ene (10.0 mmol, 2.139 g, 1.0 equiv) and the appropriate *N*-alkyl-*N,N*-dimethylamine (20.0 mmol, 2.0 equiv) were combined in acetonitrile (50 mL). The reaction mixture was heated under reflux with continuous magnetic stirring. Reaction progress was accompanied by progressive precipitation and solidification of the ionic product; solidification of the reaction mixture, preventing efficient further stirring, was used as the practical reaction endpoint. After cooling, the crude product was isolated and recrystallized from an acetonitrile/methanol mixture (10:1, v/v). The purified solid was dried at 60 °C and subsequently dried over P_4O_{10} under vacuum in a desiccator.

Compound	Dibromobutene	Tertiary amine	CH_3CN	Time	Yield	Isolated product
12-4ene-12	10.0 mmol;<br>2.139 g	20.0 mmol;<br>4.268 g	50 mL	9 h	97%	6.215 g
14-4ene-14	10.0 mmol;<br>2.139 g	20.0 mmol;<br>4.829 g	50 mL	8 h	96%	6.690 g
16-4ene-16	10.0 mmol;<br>2.139 g	20.0 mmol;<br>5.390 g	50 mL	7 h	96%	7.228 g
18-4ene-18	10.0 mmol;<br>2.139 g	20.0 mmol;<br>5.951 g	50 mL	4 h	95%	7.686 g

S2. Detailed synthesis of individual gemini surfactants

S3.1. 12-4ene-12

but-2-ene-1,4-bis(N-dodecyl-N,N-dimethylammonium bromide) ($C_{32}H_{68}Br_2N_2$, M = 640.72 g mol^{-1}). trans-1,4-Dibromobut-2-ene (10.0 mmol, 2.139 g) and N-dodecyl-N,N-dimethylamine (20.0 mmol, 4.268 g) were dissolved/suspended in acetonitrile (50 mL). The mixture was heated under reflux with magnetic stirring for 9 h, until extensive precipitation/solidification of the product prevented efficient stirring. After cooling, the crude solid was isolated, recrystallized from acetonitrile/methanol (10:1, v/v), dried at 60 °C, and then dried over P_4O_{10} under vacuum. The product was obtained in 97% isolated yield (calculated mass on the stated 10.0 mmol scale: 6.215 g) as a solid; m.p. 216–217 °C.

S3.2. 14-4ene-14

but-2-ene-1,4-bis(N-tetradecyl-N,N-dimethylammonium bromide) ($C_{36}H_{76}Br_2N_2$, M = 696.83 g mol^{-1}). trans-1,4-Dibromobut-2-ene (10.0 mmol, 2.139 g) and N-tetradecyl-N,N-dimethylamine (20.0 mmol, 4.829 g) were dissolved/suspended in acetonitrile (50 mL). The mixture was heated under reflux with magnetic stirring for 8 h, until extensive precipitation/solidification of the product prevented efficient stirring. After cooling, the crude solid was isolated, recrystallized from acetonitrile/methanol (10:1, v/v), dried at 60 °C, and then dried over P_4O_{10} under vacuum. The product was obtained in 96% isolated yield (calculated mass on the stated 10.0 mmol scale: 6.690 g) as a solid; m.p. 218–220 °C.

S3.3. 16-4ene-16

but-2-ene-1,4-bis(N-hexadecyl-N,N-dimethylammonium bromide) ($C_{40}H_{84}Br_2N_2$, M = 752.93 g mol^{-1}). trans-1,4-Dibromobut-2-ene (10.0 mmol, 2.139 g) and N-hexadecyl-N,N-dimethylamine (20.0 mmol, 5.390 g) were dissolved/suspended in acetonitrile (50 mL). The mixture was heated under reflux with magnetic stirring for 7 h, until extensive precipitation/solidification of the product prevented efficient stirring. After cooling, the crude solid was isolated, recrystallized from acetonitrile/methanol (10:1, v/v), dried at 60 °C, and then dried over P_4O_{10} under vacuum. The product was obtained in 96% isolated yield (calculated mass on the stated 10.0 mmol scale: 7.228 g) as a solid; m.p. 216–218 °C.

S3.4. 18-4ene-18

but-2-ene-1,4-bis(N-octadecyl-N,N-dimethylammonium bromide) ($C_{44}H_{92}Br_2N_2$, M = 809.04 g mol^{-1}). trans-1,4-Dibromobut-2-ene (10.0 mmol, 2.139 g) and N-octadecyl-N,N-dimethylamine (20.0 mmol, 5.951 g) were dissolved/suspended in acetonitrile (50 mL). The mixture was heated under reflux with magnetic stirring for 4 h, until extensive precipitation/solidification of the product prevented efficient stirring. After cooling, the crude solid was isolated, recrystallized from acetonitrile/methanol (10:1, v/v), dried at 60 °C, and then dried over P_4O_{10} under vacuum. The product was obtained in 95% isolated yield (calculated mass on the stated 10.0 mmol scale: 7.686 g) as a solid; m.p. 178–180 °C.

S3. NMR spectra

Insert the original spectra exported from the NMR software at sufficient resolution. Suggested order and captions are given below.

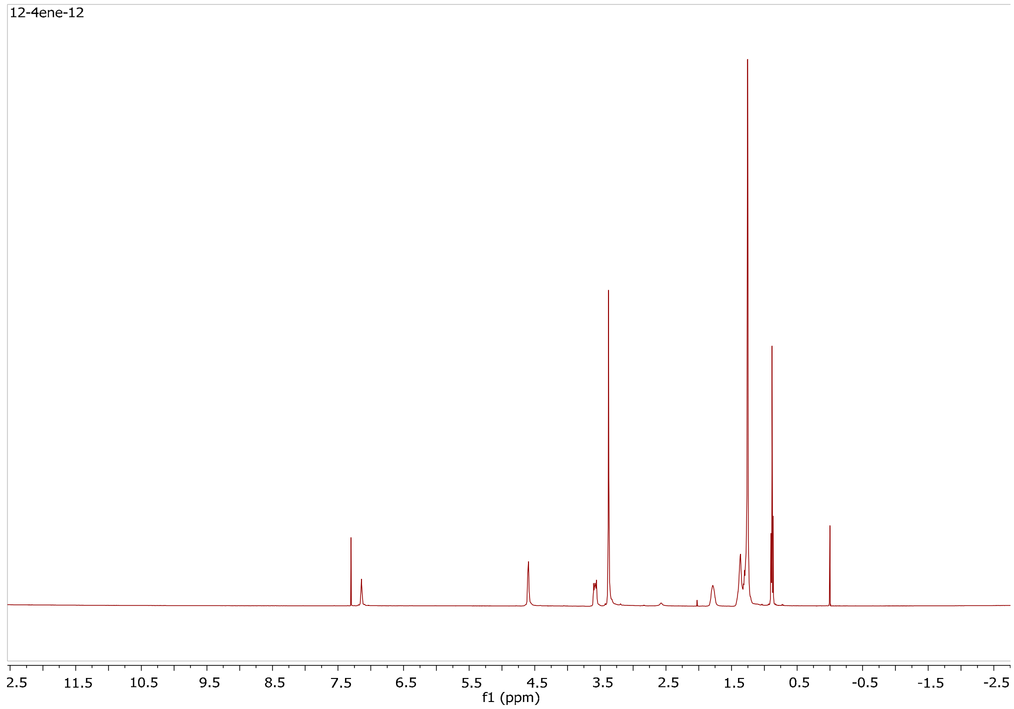

Figure S1. **^1H NMR spectrum of 12-4ene-12 (403 MHz, $CDCl_3$).**

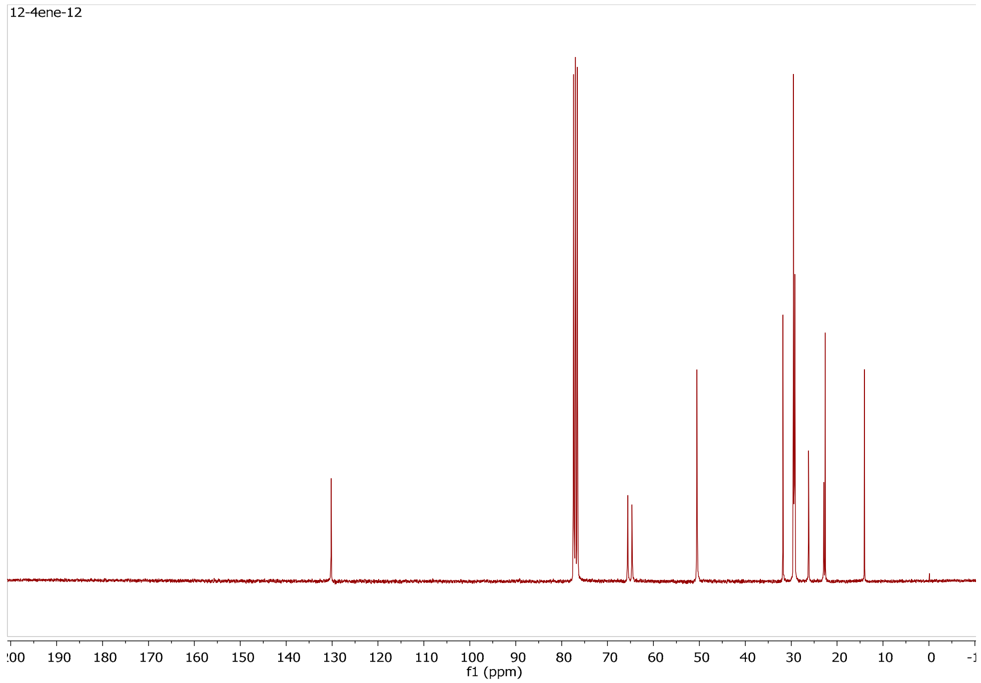

Figure S2. **^{13}C NMR spectrum of 12-4ene-12 (101 MHz, $CDCl_3$).**

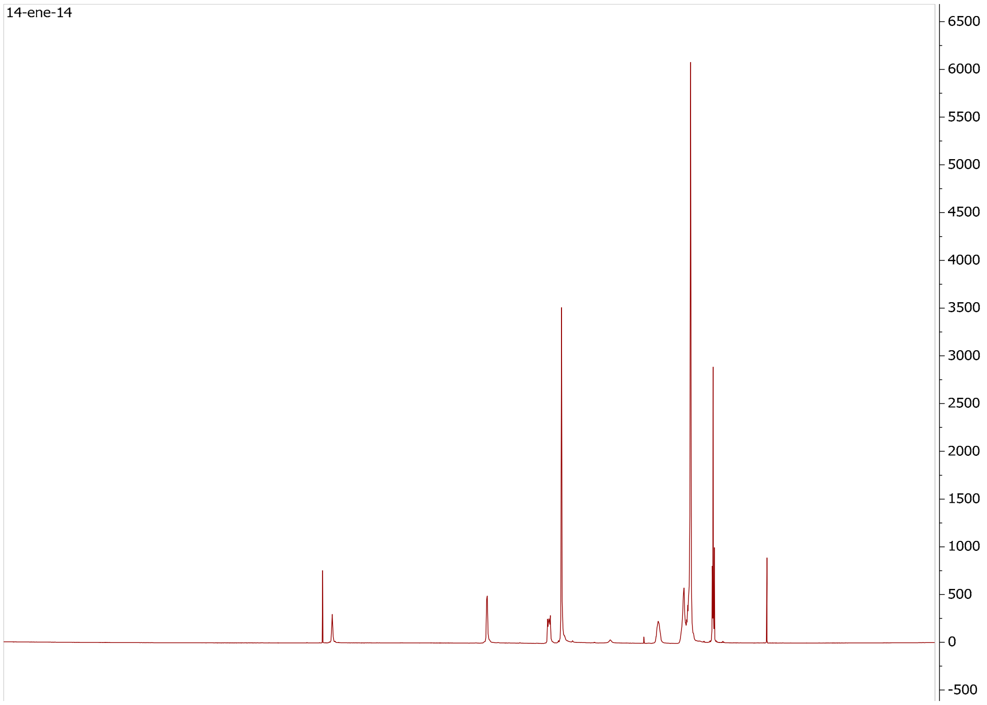

Figure S3. ^1H NMR spectrum of 14-4ene-14 (403 MHz, $CDCl_3$).

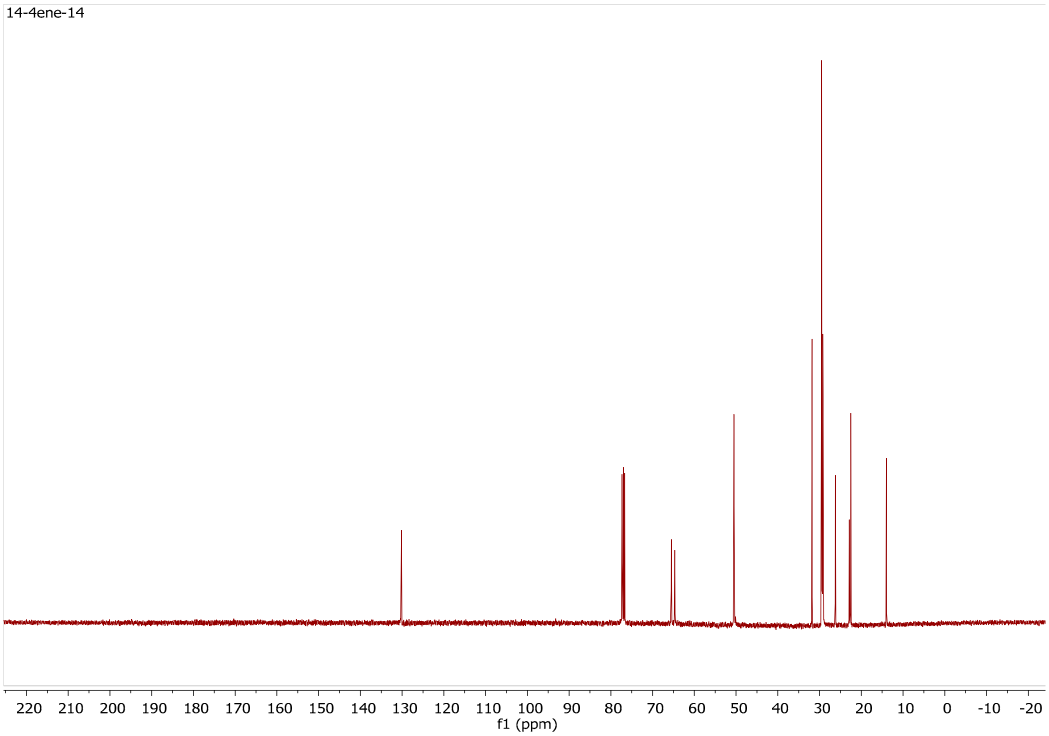

Figure S4. ^{13}C NMR spectrum of 14-4ene-14 (101 MHz, $CDCl_3$).

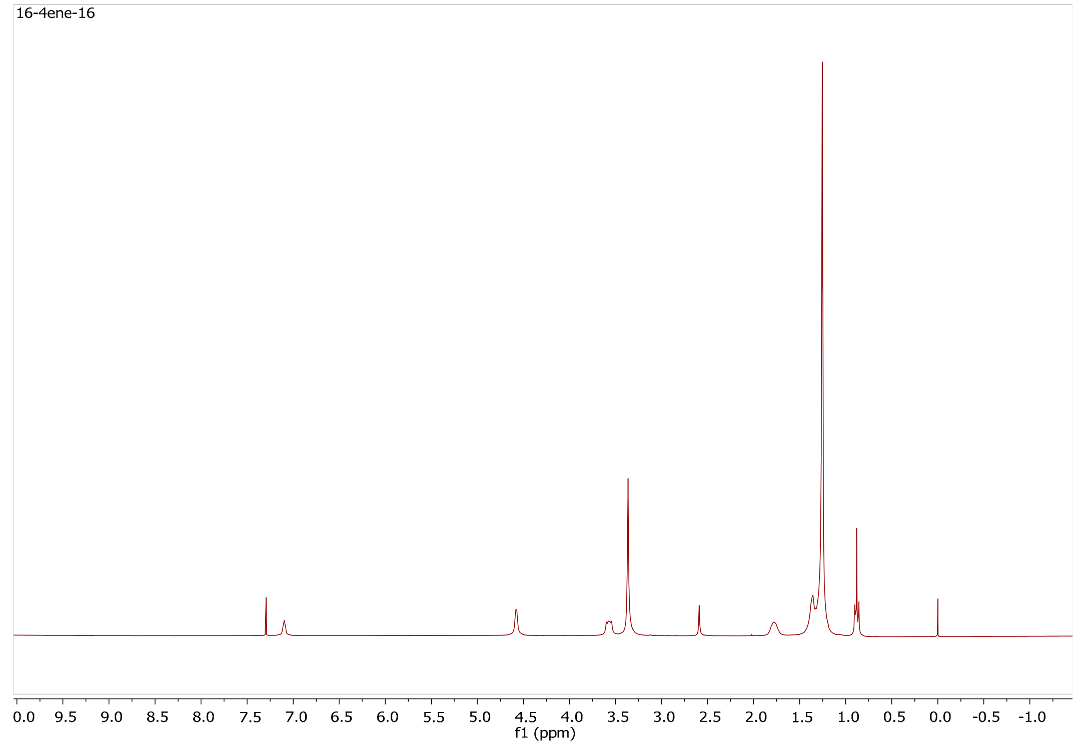

Figure S5. ¹H NMR spectrum of 16-4ene-16 (403 MHz, $CDCl_3$).

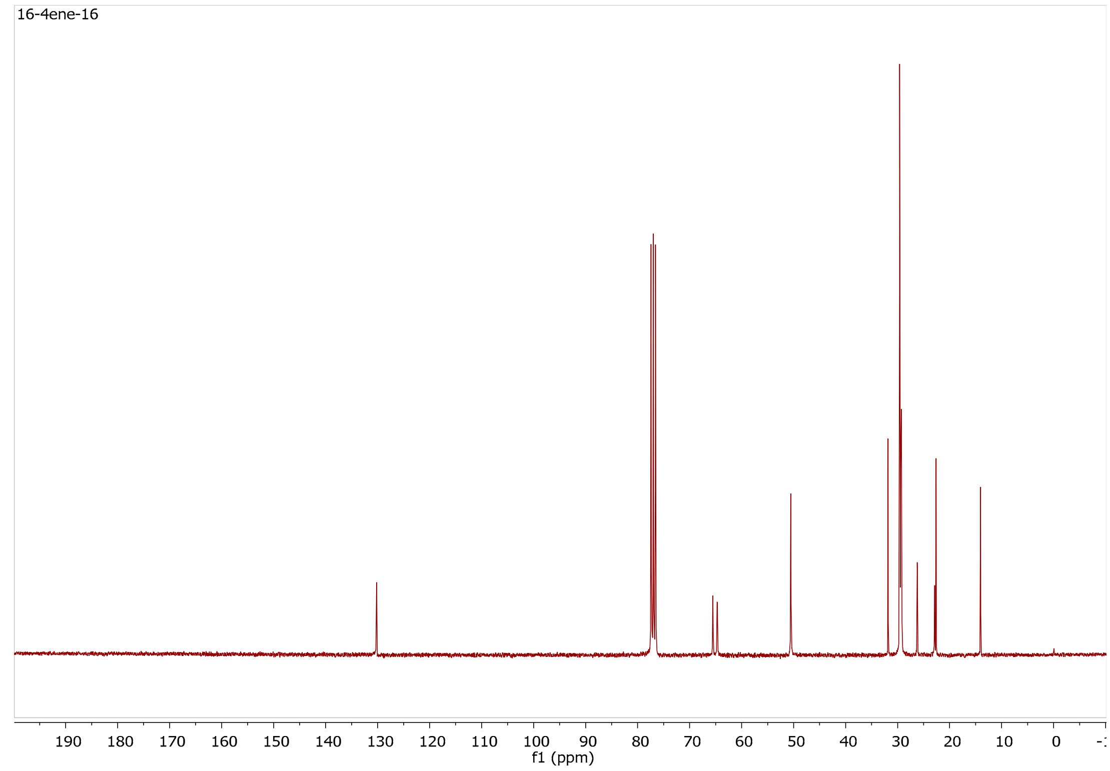

Figure S6. ¹³C NMR spectrum of 16-4ene-16 (101 MHz, $CDCl_3$).

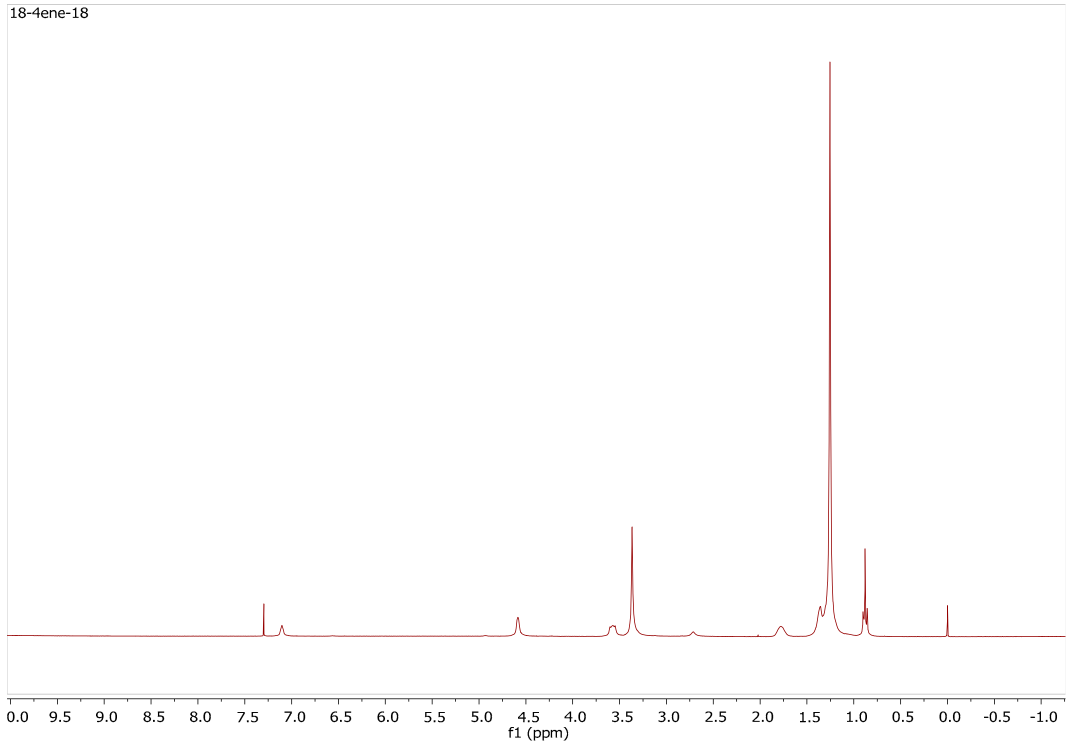

Figure S7. ^{1}H NMR spectrum of 18-4ene-18 (403 MHz, $CDCl_3$).

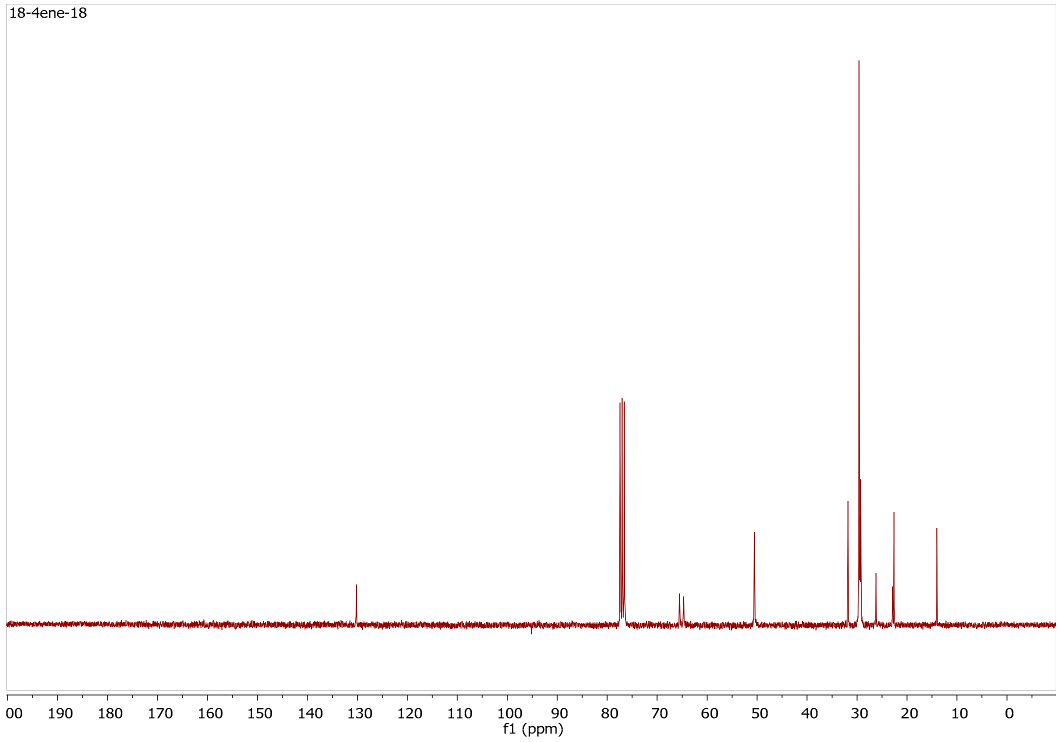

Figure S8. ^{13}C NMR spectrum of 18-4ene-18 (101 MHz, $CDCl_3$).

S4. Conductivity plots used for CMC determination

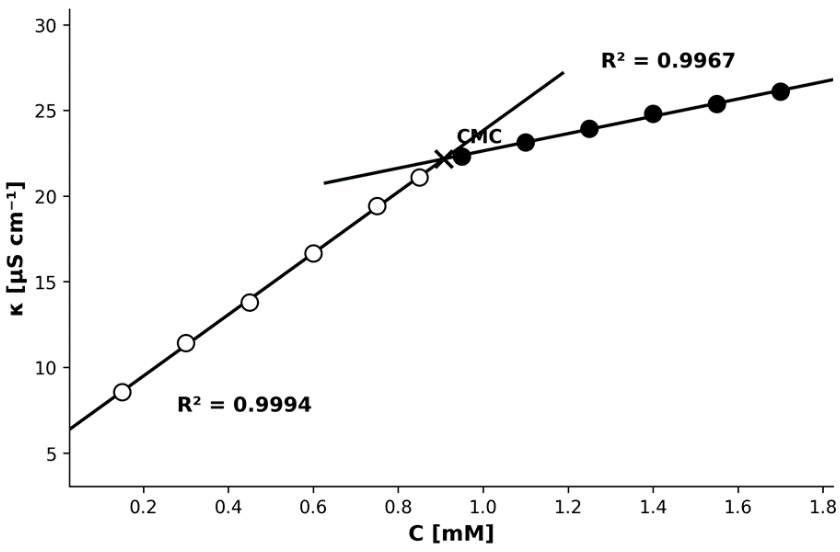

Figure S9. Conductivity plot used for CMC determination of 12-4ene-12.

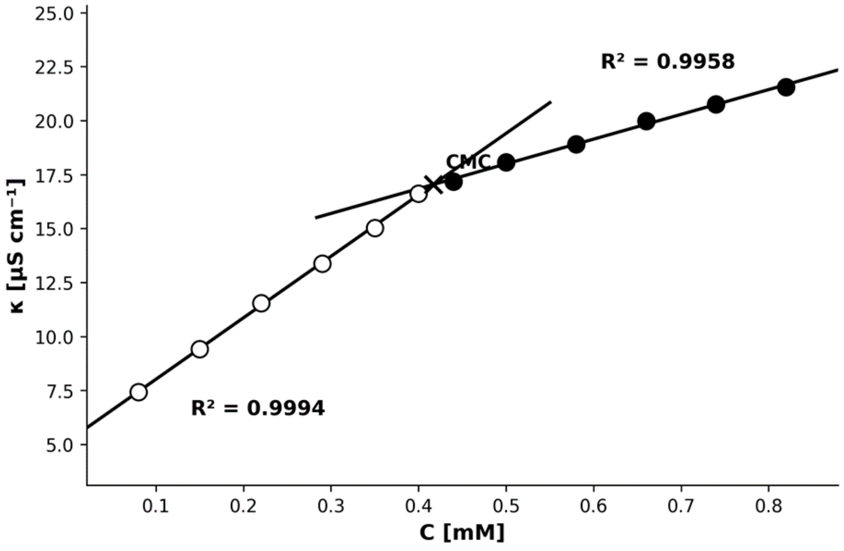

Figure S10. Conductivity plot used for CMC determination of 14-4ene-14.

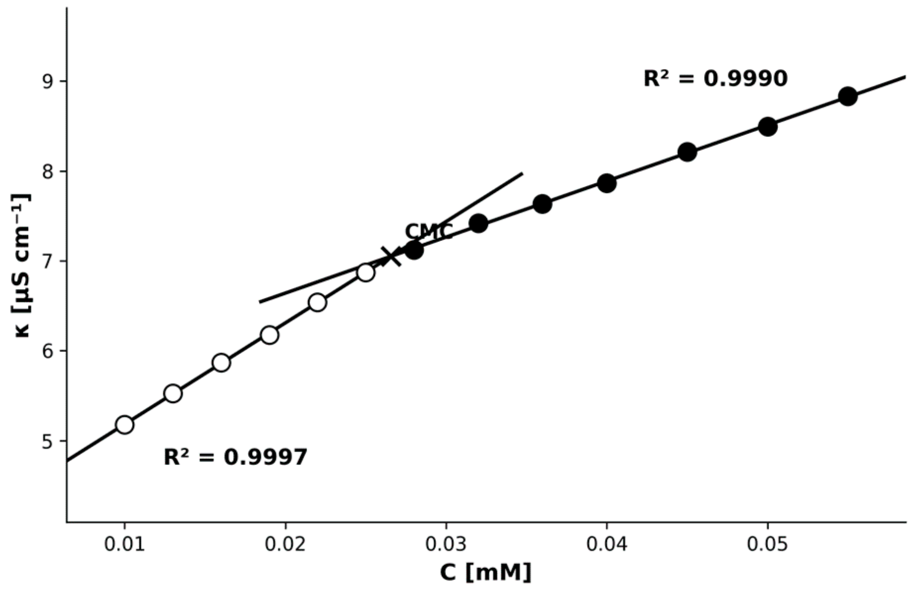

Figure S11. Conductivity plot used for CMC determination of 16-4ene-16.

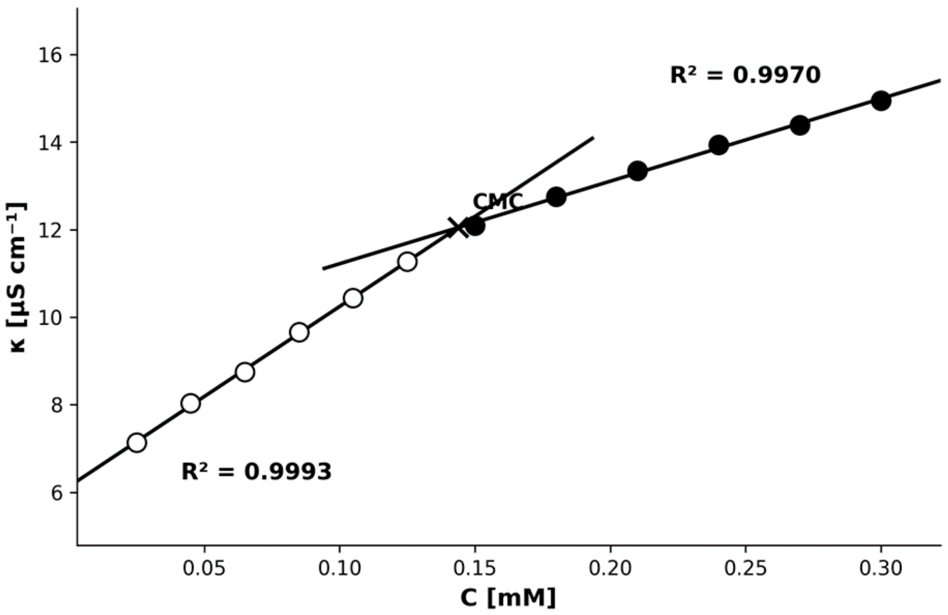

Figure S12. Conductivity plot used for CMC determination of 18-4ene-18.
